# Supplementary figures and images for: COVID-19 managed on respiratory wards and intensive care units: Results from the national COVID-19 outcome report in Wales from March 2020 to December 2021
Source: PLoS One. 2024 Jan 19;19(1):e0294895. doi: 10.1371/journal.pone.0294895 (PMC10798461; doi:10.1371/journal.pone.0294895)

**S2 Figure. Length of stay, whole cohort and treatment subgroups**

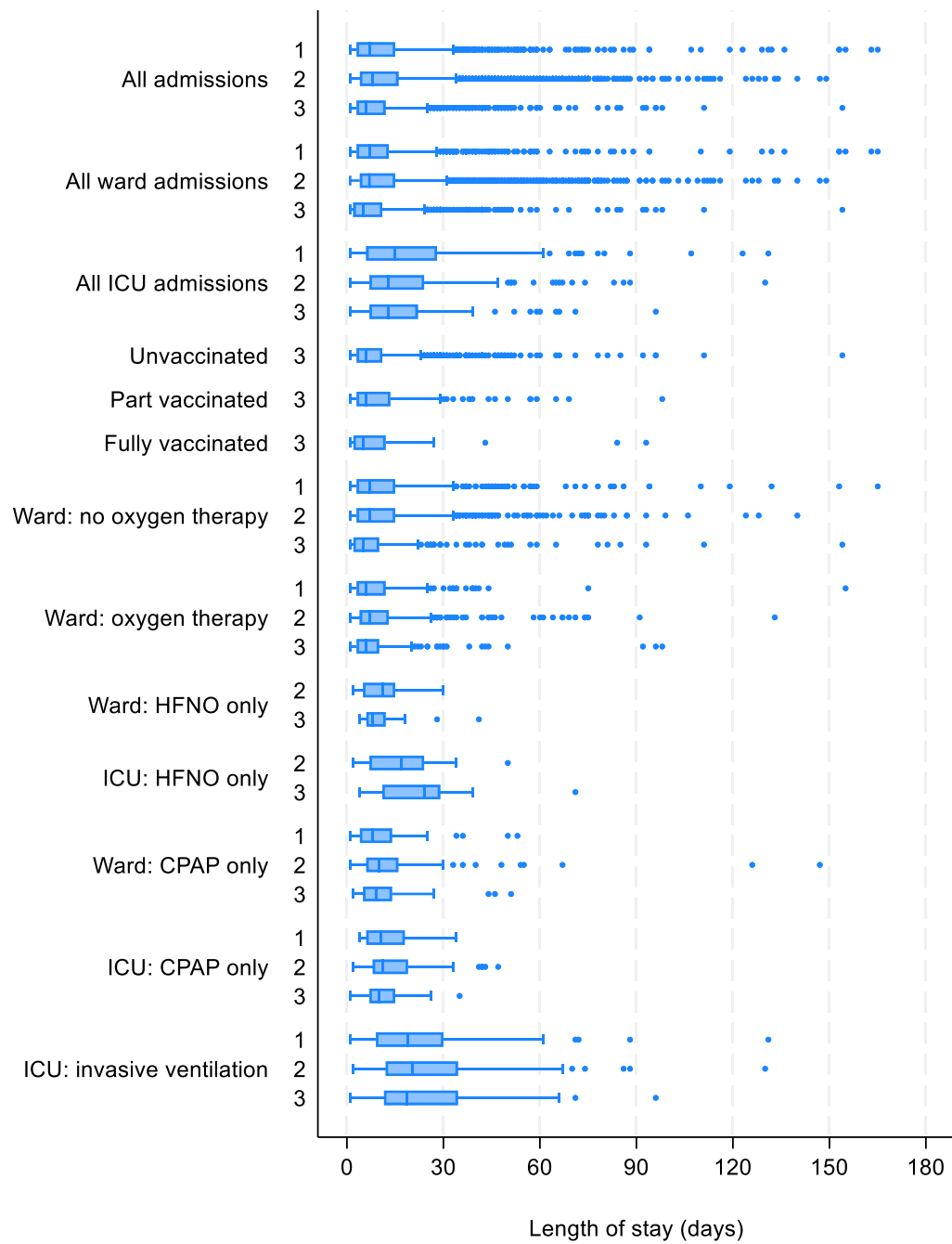

Supplement: S1 Fig — (PDF) [file pone.0294895.s001.pdf]

**S8 Figure. Univariate logistic regression, whole cohort**

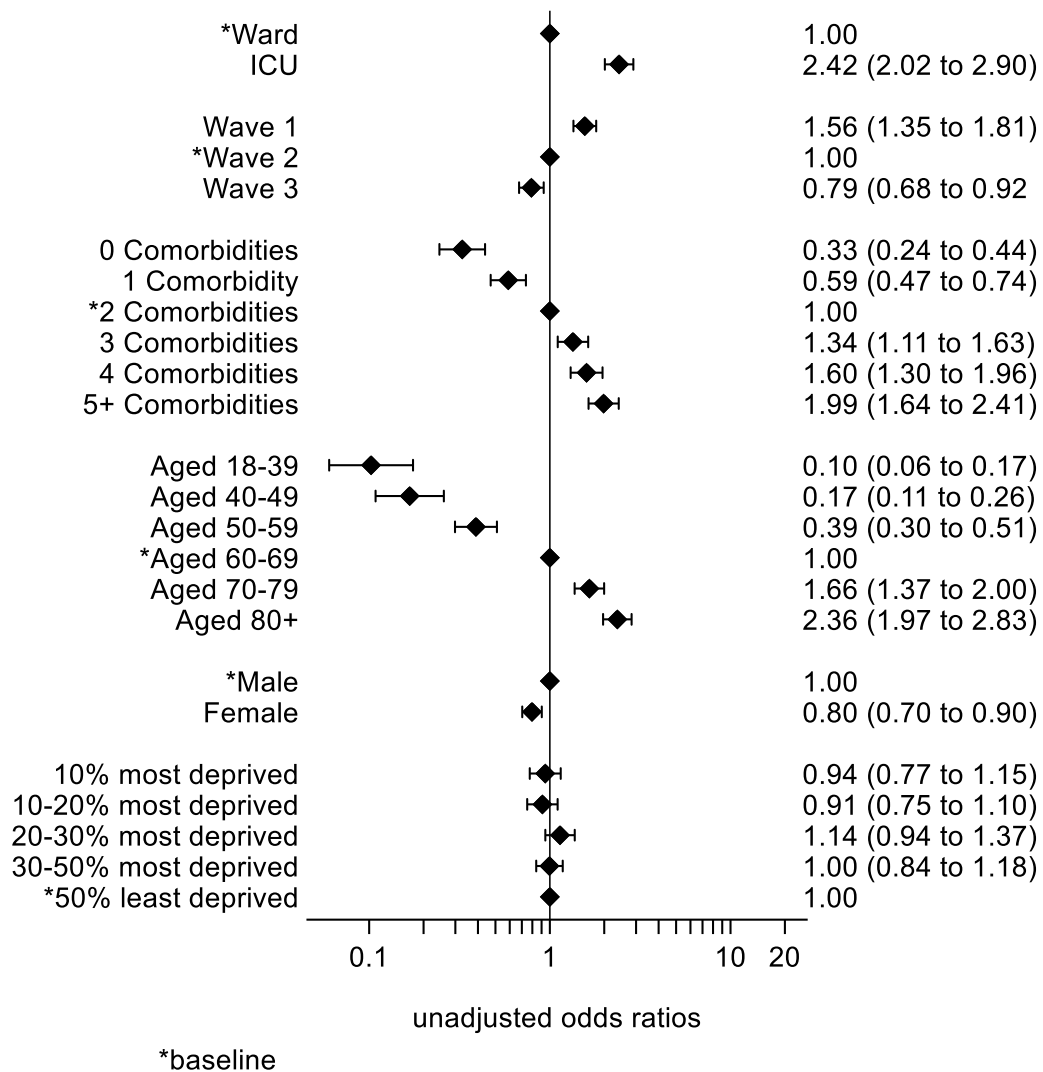

Supplement: S2 Fig — (PDF) [file pone.0294895.s002.pdf]

**S20 Figure. Univariate logistic regression, CPAP subgroup**

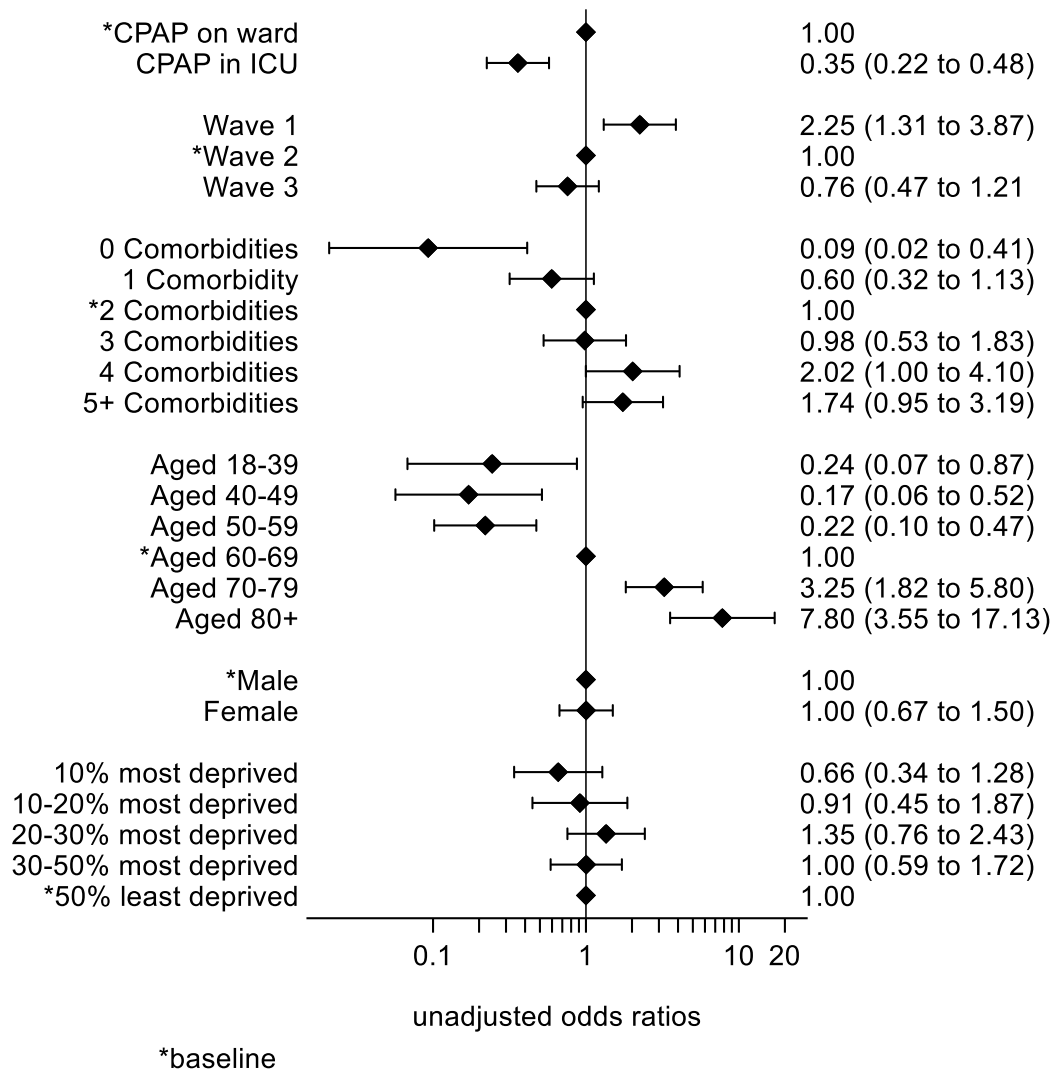

Supplement: S3 Fig — (PDF) [file pone.0294895.s003.pdf]
