# Supplementary material for: COVID-19 managed on respiratory wards and intensive care units: Results from the national COVID-19 outcome report in Wales from March 2020 to December 2021
Source: PLoS One. 2024 Jan 19;19(1):e0294895. doi: 10.1371/journal.pone.0294895 (PMC10798461; doi:10.1371/journal.pone.0294895)
Supplement: S1 Table — (PDF) [file pone.0294895.s004.pdf]

S1 Table. Non-mandatory data completeness, whole cohort

Non-mandatory fields (% recorded)	Clinical Frailty Score 65+			Comorbidity count			Vaccine status			Obesity (% yes)		
Wave	1	2	3	1	2	3	1	2	3	1	2	3
Health Board 1	58.2	0.1	2.7	100.0	100.0	100.0	n/a	n/a	100.0	12.6	7.8	10.8
Health Board 2	22.5	3.2	0.0	100.0	100.0	100.0	n/a	n/a	100.0	7.8	13.9	8.3
Health Board 3	61.6	84.3	75.4	100.0	100.0	100.0	n/a	n/a	100.0	8.6	20.7	25.8
Health Board 4	68.2	74.9	97.2	100.0	100.0	100.0	n/a	n/a	100.0	7.3	9.6	13.0
Health Board 5	43.6	66.7	21.6	100.0	100.0	100.0	n/a	n/a	100.0	6.1	6.9	6.4
Health Board 6	70.7	49.0	7.7	100.0	100.0	100.0	n/a	n/a	100.0	8.8	11.7	6.5
All Health Boards	58.4	72.2	90.1	100.0	100.0	100.0	n/a	n/a	100.0	8.9	10.5	11.6

Treatment fields (% yes)	Oxygen			Dexamethasone			Remdesivir			CPAP/HFNO			IL-6		
Wave	1	2	3	1	2	3	1	2	3	1	2	3	1	2	3
Health Board 1	67.7	2.0	11.2	0.0	0.4	9.4	0.0	0.0	0.0	12.6	1.7	0.9	0.0	0.0	1.5
Health Board 2	72.6	90.8	58.9	2.0	87.2	61.3	0.7	36.4	15.5	20.3	55.4	21.4	0.0	24.6	4.8
Health Board 3	59.9	61.9	71.1	1.8	62.6	68.4	1.4	35.6	27.9	10.8	10.1	30.5	0.0	3.8	33.7
Health Board 4	53.5	77.9	66.3	1.3	73.1	61.0	0.2	2.0	0.0	9.9	25.7	20.3	0.0	1.6	17.5
Health Board 5	45.9	59.1	45.7	1.0	55.8	49.5	1.0	8.8	1.6	12.2	13.3	13.9	0.0	1.1	16.3
Health Board 6	68.6	77.7	54.8	0.0	73.9	51.6	0.0	50.5	22.6	2.9	16.5	9.7	0.0	0.5	0.0
All Health Boards	61.0	71.6	58.0	1.0	68.7	57.9	0.4	39.4	22.1	11.3	21.8	19.7	0.0	2.7	11.9
