# Supplementary material for: COVID-19 managed on respiratory wards and intensive care units: Results from the national COVID-19 outcome report in Wales from March 2020 to December 2021
Source: PLoS One. 2024 Jan 19;19(1):e0294895. doi: 10.1371/journal.pone.0294895 (PMC10798461; doi:10.1371/journal.pone.0294895)
Supplement: S2 Table — (PDF) [file pone.0294895.s005.pdf]

S2 Table. Whole cohort counts and percents

		All admissions			All ward admissions			All ICU admissions		
		Wave 1	Wave 2	Wave 3	Wave 1	Wave 2	Wave 3	Wave 1	Wave 2	Wave 3
		n (%)	n (%)	n (%)	n (%)	n (%)	n (%)	n (%)	n (%)	n (%)
Age	18-39	91 (6.4)	229 (7.8)	229 (14.8)	74 (6.2)	211 (7.9)	218 (15.3)	17 (7.2)	18 (7.7)	11 (9.3)
	40-49	120 (8.4)	276 (9.5)	158 (10.2)	99 (8.3)	245 (9.1)	138 (9.7)	21 (8.9)	31 (13.2)	20 (16.9)
	50-59	228 (16.0)	447 (15.3)	215 (13.9)	169 (14.2)	386 (14.4)	184 (12.9)	59 (25.1)	61 (26.0)	31 (26.3)
	60-69	273 (19.2)	518 (17.7)	282 (18.3)	203 (17.1)	447 (16.7)	248 (17.4)	70 (29.8)	71 (30.2)	34 (28.8)
	70-79	344 (24.2)	688 (23.6)	319 (20.7)	295 (24.8)	653 (24.3)	303 (21.2)	49 (20.9)	35 (14.9)	16 (13.6)
	80+	368 (25.8)	761 (26.1)	341 (22.1)	349 (29.4)	742 (27.6)	335 (23.5)	19 (8.1)	19 (8.1)	6 (5.1)
	All	1,424 (100)	2,919 (100)	1,544 (100)	1,189 (100)	2,684 (100)	1,426 (100)	235 (100)	235 (100)	118 (100)
Sex	Male	810 (56.9)	1,586 (54.3)	844 (54.7)	657 (55.3)	1,431 (53.3)	769 (53.9)	153 (65.1)	155 (66.0)	75 (63.6)
	Female	614 (43.1)	1333 (45.7)	700 (45.3)	532 (44.7)	1,253 (46.7)	657 (46.1)	82 (34.9)	80 (34.0)	43 (36.4)
	All	1,424 (100)	2,919 (100)	1,544 (100)	1,189 (100)	2,684 (100)	1,426 (100)	235 (100)	235 (100)	118 (100)
Comorbidities	0	193 (13.6)	401 (13.7)	189 (12.2)	162 (13.6)	369 (13.7)	175 (12.3)	31 (13.2)	32 (13.6)	14 (11.9)
	1	291 (20.4)	479 (16.4)	271 (17.6)	237 (19.9)	417 (15.5)	253 (17.7)	54 (23.0)	62 (26.4)	18 (15.3)
	2	286 (20.1)	647 (22.2)	288 (18.7)	234 (19.7)	605 (22.5)	258 (18.1)	52 (22.1)	42 (17.9)	30 (25.4)
	3	264 (18.5)	534 (18.3)	270 (17.5)	219 (18.4)	495 (18.4)	245 (17.2)	45 (19.1)	39 (16.6)	25 (21.2)
	4	177 (12.4)	427 (14.6)	215 (13.9)	154 (13.0)	407 (15.2)	201 (14.1)	23 (9.8)	20 (8.5)	14 (11.9)
	5+	213 (15.0)	431 (14.8)	311 (20.1)	183 (15.4)	391 (14.6)	294 (20.6)	30 (12.8)	40 (17.0)	17 (14.4)
	All	1,424 (100)	2,919 (100)	1,544 (100)	1,189 (100)	2,684 (100)	1,426 (100)	235 (100)	235 (100)	118 (100)
Deprivation	most 10%	184 (13.6)	370 (13.4)	192 (12.8)	149 (13.2)	344 (13.6)	181 (13.0)	35 (15.8)	26 (11.8)	11 (9.6)
	most 10-20%	165 (12.2)	412 (15.0)	217 (14.4)	134 (11.8)	394 (15.5)	205 (14.8)	31 (14.0)	18 (8.2)	12 (10.4)
	most 20-30%	206 (15.2)	356 (12.9)	214 (14.2)	171 (15.1)	320 (12.6)	189 (13.6)	35 (15.8)	36 (16.4)	25 (21.7)
	most 30-50%	268 (19.8)	537 (19.5)	321 (21.4)	233 (20.6)	493 (19.5)	298 (21.5)	35 (15.8)	44 (20.0)	23 (20.0)
	least 50%	531 (39.2)	1079 (39.2)	559 (37.2)	446 (39.4)	983 (38.8)	515 (37.1)	85 (38.5)	96 (43.6)	44 (38.3)
	All	1,354 (100)	2,754 (100)	1,503 (100)	1,133 (100)	2,534 (100)	1,388 (100)	221 (100)	220 (100)	115 (100)
