# Supplementary material for: COVID-19 managed on respiratory wards and intensive care units: Results from the national COVID-19 outcome report in Wales from March 2020 to December 2021
Source: PLoS One. 2024 Jan 19;19(1):e0294895. doi: 10.1371/journal.pone.0294895 (PMC10798461; doi:10.1371/journal.pone.0294895)
Supplement: S3 Table — (PDF) [file pone.0294895.s006.pdf]

S3 Table. Length of stay, whole cohort and treatment subgroups

	Wave 1 Median (IQR)		Wave 2 Median (IQR)		Wave 3 Median (IQR)	
All admissions	7	(3 to 15)	8	(4 to 16)	6	(3 to 12)
All ward admissions	7	(3 to 13)	7	(4 to 15)	5	(2 to 11)
All ICU admissions	15	(6 to 28)	13	(7 to 24)	13	(7 to 22)
Unvaccinated	-	-	-	-	6	(3 to 11)
Part vaccinated	-	-	-	-	6	(3 to 13.5)
Fully vaccinated	-	-	-	-	5	(2 to 12)
Subgroups, excluding HB1:						
Ward, without O2 therapy	7	(3 to 15)	7	(3 to 15)	5	(2 to 10)
Ward, with O2 therapy	6	(3 to 12)	7	(4 to 13)	6	(3 to 10)
Ward, with HFNO only	-	-	11	(5 to 15)	8	(6 to 12)
ICU, with HFNO only	-	-	17	(7 to 24)	24	(11 to 29)
Ward, with CPAP only	8	(4 to 14)	10	(6 to 17)	9	(5 to 14)
ICU, with CPAP only	10.5	(6 to 18)	11	(8 to 19)	10	(7 to 15)
ICU, with invasive ventilation	19	(9 to 30)	20.5	(12 to 34.5)	18.5	(11.5 to 34.5)
