# Supplementary material for: COVID-19 managed on respiratory wards and intensive care units: Results from the national COVID-19 outcome report in Wales from March 2020 to December 2021
Source: PLoS One. 2024 Jan 19;19(1):e0294895. doi: 10.1371/journal.pone.0294895 (PMC10798461; doi:10.1371/journal.pone.0294895)
Supplement: S4 Table — (PDF) [file pone.0294895.s007.pdf]

S5 Table. Wave 3 vaccination status counts and percents

		Unvaccinated n (%)	Part vaccinated n (%)	Fully vaccinated n (%)
Age	18-39	196 (16.5)	29 (10.7)	4 (4.8)
	40-49	117 (9.8)	34 (12.5)	7 (8.4)
	50-59	162 (13.6)	39 (14.3)	14 (16.9)
	60-69	215 (18.1)	53 (19.5)	14 (16.9)
	70-79	230 (19.3)	69 (25.4)	20 (24.1)
	80+	269 (22.6)	48 (17.6)	24 (28.9)
	All	1,189 (100)	272 (100)	83 (100)
Sex	Male	655 (55.1)	146 (53.7)	43 (51.8)
	Female	534 (44.9)	126 (46.3)	40 (48.2)
	All	1,189 (100)	272 (100)	83 (100)
Comorbidities	0	166 (14.0)	19 (7.0)	4 (4.8)
	1	209 (17.6)	47 (17.3)	15 (18.1)
	2	210 (17.7)	69 (25.4)	9 (10.8)
	3	205 (17.2)	52 (19.1)	13 (15.7)
	4	171 (14.4)	36 (13.2)	8 (9.6)
	5+	228 (19.2)	49 (18.0)	34 (41.0)
	All	1,189 (100)	272 (100)	83 (100)
Deprivation	most 10%	156 (13.4)	29 (11.2)	7 (8.9)
	most 10-20%	178 (15.3)	28 (10.8)	11 (13.9)
	most 20-30%	166 (14.3)	35 (13.5)	13 (16.5)
	most 30-50%	254 (21.8)	50 (19.2)	17 (21.5)
	least 50%	410 (35.2)	118 (45.4)	31 (39.2)
	All	1,164 (100)	260 (100)	79 (100)
