# Supplementary material for: COVID-19 managed on respiratory wards and intensive care units: Results from the national COVID-19 outcome report in Wales from March 2020 to December 2021
Source: PLoS One. 2024 Jan 19;19(1):e0294895. doi: 10.1371/journal.pone.0294895 (PMC10798461; doi:10.1371/journal.pone.0294895)
Supplement: S5 Table — (PDF) [file pone.0294895.s008.pdf]

S6 Table. Whole cohort crude outcomes

	Wave	Cases	Deaths			p value
		n	n	%	(95% CI)	
All admissions	1	1,424	448	31.5	(29.1 to 33.9)	1 v 2: p<0.01
	2	2,919	660	22.6	(21.1 to 24.2)	2 v 3: p<0.01
	3	1,544	290	18.8	(16.9 to 20.8)	3 v 1: p<0.01
All Ward admissions	1	1,189	344	28.9	(26.4 to 31.6)	1 v 2: p<0.01
	2	2,684	566	21.1	(19.6 to 22.7)	2 v 3: p<0.01
	3	1,426	252	17.7	(15.8 to 19.7)	3 v 1: p<0.01
All ICU admissions	1	235	104	44.3	(38.0 to 50.6)	1 v 2: p=0.35
	2	235	94	40.0	(33.9 to 46.4)	2 v 3: p=0.15
	3	118	38	32.2	(24.4 to 41.1)	3 v 1: p=0.03
Unvaccinated	3	1,189	244	20.5	(18.3 to 22.9)	Un v Part: p=0.06
Partly vaccinated	3	272	42	15.4	(11.6 to 20.2)	Part v Whole: p=0.02
Wholly vaccinated	3	83	4	4.8	(1.8 to 11.7)	Whole v Un: p<0.01
