# Supplementary material for: COVID-19 managed on respiratory wards and intensive care units: Results from the national COVID-19 outcome report in Wales from March 2020 to December 2021
Source: PLoS One. 2024 Jan 19;19(1):e0294895. doi: 10.1371/journal.pone.0294895 (PMC10798461; doi:10.1371/journal.pone.0294895)
Supplement: S6 Table — (PDF) [file pone.0294895.s009.pdf]

S7 Table. Univariate logistic regression, whole cohort

Variable		Coefficient (β)	SE	Wald χ^2	P value	Odds Ratio	95% CI
Setting	ICU	0.883	0.093	9.5	<0.01	2.42	2.02 to 2.90
	(Baseline) Ward	0.000				1.00	
Wave	1	0.446	0.074	6.0	<0.01	1.56	1.35 to 1.81
	(Baseline) 2	0.000				1.00	
	3	-0.235	0.080	-2.9	<0.01	0.79	0.68 to 0.92
Comorbidities	0	-1.117	0.148	-7.5	<0.01	0.33	0.24 to 0.44
	1	-0.530	0.115	-4.6	<0.01	0.59	0.47 to 0.74
	(Baseline) 2	0.000				1.00	
	3	0.295	0.099	3.0	<0.01	1.34	1.11 to 1.63
	4	0.468	0.104	4.5	<0.01	1.60	1.30 to 1.96
	5+	0.686	0.098	7.0	<0.01	1.99	1.64 to 2.41
Age	18-39	-2.278	0.273	-8.4	<0.01	0.10	0.06 to 0.17
	40-49	-1.786	0.222	-8.0	<0.01	0.17	0.11 to 0.26
	50-59	-0.942	0.136	-6.9	<0.01	0.39	0.30 to 0.51
	(Baseline) 60-69	0.000				1.00	
	70-79	0.504	0.096	5.3	<0.01	1.66	1.37 to 2.00
	80+	0.860	0.093	9.3	<0.01	2.36	1.97 to 2.83
Sex	Female	-0.227	0.063	-3.6	<0.01	0.80	0.70 to 0.90
	(Baseline) Male	0.000				1.00	
Deprivation	Most 10%	-0.059	0.101	-0.6	0.56	0.94	0.77 to 1.15
	Most 10-20%	-0.095	0.099	-1.0	0.34	0.91	0.75 to 1.10
	Most 20-30%	0.130	0.096	1.4	0.18	1.14	0.94 to 1.37
	Most 30-50%	-0.005	0.086	-0.1	0.96	1.00	0.84 to 1.18
	(Baseline) Least 50%	0.000				1.00	
