# Supplementary material for: COVID-19 managed on respiratory wards and intensive care units: Results from the national COVID-19 outcome report in Wales from March 2020 to December 2021
Source: PLoS One. 2024 Jan 19;19(1):e0294895. doi: 10.1371/journal.pone.0294895 (PMC10798461; doi:10.1371/journal.pone.0294895)
Supplement: S7 Table — (PDF) [file pone.0294895.s010.pdf]

S9 Table. Multivariable logistic regression model, whole cohort

Variable		Coefficient (β)	SE	Wald χ^2	P value	Odds Ratio	95% CI
Setting	ICU	1.485	0.112	13.2	<0.01	4.41	3.54 to 5.50
	(Baseline) Ward	0.000				1.00	
Wave	1	0.373	0.082	4.6	<0.01	1.45	1.24 to 1.71
	(Baseline) 2	0.000				1.00	
	3	-0.172	0.087	-2.0	0.05	0.84	0.71 to 1.00
Comorbidities	0	-0.715	0.158	-4.5	<0.01	0.49	0.09 to 0.26
	1	-0.385	0.124	-3.1	<0.01	0.68	0.53 to 0.87
	(Baseline) 2	0.000				1.00	
	3	0.189	0.106	1.8	0.08	1.21	0.98 to 1.49
	4	0.340	0.111	3.1	<0.01	1.41	1.13 to 1.75
	5+	0.442	0.106	4.2	<0.01	1.56	1.26 to 1.91
Age	18-39	-1.890	0.279	-6.8	<0.01	0.15	0.09 to 0.26
	40-49	-1.693	0.228	-7.4	<0.01	0.18	0.12 to 0.29
	50-59	-0.956	0.143	-6.7	<0.01	0.38	0.29 to 0.51
	(Baseline) 60-69	0.000				1.00	
	70-79	0.590	0.103	5.8	<0.01	1.80	1.48 to 2.21
	80+	1.055	0.101	10.5	<0.01	2.87	2.36 to 3.50
Sex	Female	-0.194	0.070	-2.8	0.01	0.82	0.72 to 0.95
	(Baseline) Male	0.000				1.00	
Deprivation	Most 10%	0.157	0.111	1.4	0.16	1.17	0.94 to 1.45
	Most 10-20%	0.040	0.108	0.4	0.71	1.04	0.84 to 1.29
	Most 20-30%	0.190	0.106	1.8	0.07	1.21	0.98 to 1.49
	Most 30-50%	0.091	0.094	1.0	0.33	1.10	0.91 to 1.32
	(Baseline) Least 50%	0.000				1.00	
	Constant	-1.638	0.122				
