# Supplementary material for: COVID-19 managed on respiratory wards and intensive care units: Results from the national COVID-19 outcome report in Wales from March 2020 to December 2021
Source: PLoS One. 2024 Jan 19;19(1):e0294895. doi: 10.1371/journal.pone.0294895 (PMC10798461; doi:10.1371/journal.pone.0294895)
Supplement: S8 Table — (PDF) [file pone.0294895.s011.pdf]

S12 Table. Subgroup summary statistics: oxygen therapy

	Wave	No oxygen therapy		Oxygen therapy	
		**Median**	**IQR**	**Median**	**IQR**
Age	1	71	57 to 81	71.5	54 to 82
	2	70	52 to 82	69	55 to 78
	3	67	42 to 80	67	50 to 78
	All	69	52 to 81	69	54 to 79
Comorbidities	1	2	1 to 3	2	1 to 4
	2	2	1 to 4	2	1 to 4
	3	2	1 to 4	2	1 to 4
	All	2	1 to 4	2	1 to 4
		**30% most**	**50% least**	**30% most**	**50% least**
Deprivation (% from areas in most deprived 30% and least deprived 50%)	1	41.5	38.3	36.9	43.5
	2	39.7	42.1	39.8	41.4
	3	35.9	40.9	39.6	37.3
	All	39.2	40.1	39.0	40.8
		**Male**	**Female**	**Male**	**Female**
Sex (% of each sex receiving / not receiving the treatment)	1	65.6	70.7	34.4	29.3
	2	55.8	63.3	44.2	36.7
	3	59.4	69.3	40.6	30.7
	All	59.7	67.0	40.3	33.0
