# Supplementary material for: COVID-19 managed on respiratory wards and intensive care units: Results from the national COVID-19 outcome report in Wales from March 2020 to December 2021
Source: PLoS One. 2024 Jan 19;19(1):e0294895. doi: 10.1371/journal.pone.0294895 (PMC10798461; doi:10.1371/journal.pone.0294895)
Supplement: S9 Table — (PDF) [file pone.0294895.s012.pdf]

S13 Table. Subgroup counts and percents: oxygen therapy

		Wave 1		Wave 2		Wave 3	
		No O2	O2 given	No O2	O2 given	No O2	O2 given
		n (%)	n (%)	n (%)	n (%)	n (%)	n (%)
Age	18-39	38 (73.1)	14 (26.9)	72 (72.0)	28 (28.0)	104 (76.5)	32 (23.5)
	40-49	50 (62.5)	30 (37.5)	92 (59.0)	64 (41.0)	47 (56.0)	37 (44.0)
	50-59	78 (63.4)	45 (36.6)	100 (54.9)	82 (45.1)	55 (60.4)	36 (39.6)
	60-69	102 (72.9)	38 (27.1)	109 (52.7)	98 (47.3)	67 (54.5)	56 (45.5)
	70-79	132 (71.0)	54 (29.0)	157 (54.0)	134 (46.0)	98 (64.5)	54 (35.5)
	80+	167 (65.7)	87 (34.3)	230 (67.3)	112 (32.7)	126 (66.3)	64 (33.7)
	All	567 (67.9)	268 (32.1)	760 (59.5)	518 (40.5)	497 (64.0)	279 (36.0)
Sex	Male	295 (65.6)	155 (34.4)	365 (55.8)	289 (44.2)	244 (59.4)	167 (40.6)
	Female	272 (70.6)	113 (29.4)	395 (63.3)	229 (36.7)	253 (69.3)	112 (30.7)
	All	567 (67.9)	268 (32.1)	760 (59.5)	518 (40.5)	497 (64.0)	279 (36.0)
Comorbidities	0	108 (77.1)	32 (22.9)	106 (66.7)	53 (33.3)	73 (66.4)	37 (33.6)
	1	121 (69.9)	52 (30.1)	156 (61.4)	98 (38.6)	98 (62.8)	58 (37.2)
	2	104 (62.7)	62 (37.3)	159 (54.6)	132 (45.4)	90 (62.1)	55 (37.9)
	3	102 (67.5)	49 (32.5)	113 (56.5)	87 (43.5)	85 (65.9)	44 (34.1)
	4	54 (57.4)	40 (42.6)	117 (62.9)	69 (37.1)	64 (67.4)	31 (32.6)
	5+	78 (70.3)	33 (29.7)	109 (58.0)	79 (42.0)	87 (61.7)	54 (38.3)
	All	567 (67.9)	268 (32.1)	760 (59.5)	518 (40.5)	497 (64.0)	279 (36.0)
Deprivation	most 10%	56 (56.6)	43 (43.4)	75 (50.7)	73 (49.3)	53 (63.1)	31 (36.9)
	most 10-20%	59 (74.7)	20 (25.3)	88 (60.3)	58 (39.7)	52 (60.5)	34 (39.5)
	most 20-30%	107 (77.5)	31 (22.5)	113 (68.9)	51 (31.1)	67 (62.0)	41 (38.0)
	most 30-50%	108 (68.4)	50 (31.6)	127 (59.6)	86 (40.4)	111 (64.2)	62 (35.8)
	least 50%	205 (64.9)	111 (35.1)	293 (60.8)	189 (39.2)	196 (66.2)	100 (33.8)
	All	535 (67.7)	255 (32.3)	696 (60.4)	457 (39.6)	479 (64.1)	268 (35.9)
