# Supplementary material for: COVID-19 managed on respiratory wards and intensive care units: Results from the national COVID-19 outcome report in Wales from March 2020 to December 2021
Source: PLoS One. 2024 Jan 19;19(1):e0294895. doi: 10.1371/journal.pone.0294895 (PMC10798461; doi:10.1371/journal.pone.0294895)
Supplement: S10 Table — (PDF) [file pone.0294895.s013.pdf]

S14 Table. Subgroup summary statistics: HFNO only

	Wave	Ward HFNO admissions		ICU HFNO admissions	
		Median	IQR	Median	IQR
Age	1	-	-	-	-
	2	65	58 to 72	52	43 to 64
	3	71	66 to 81	55	50 to 69
	All	68	59.5 to 77	54	43 to 69
Comorbidities	1	-	-	-	-
	2	3	1 to 3	3	1 to 4
	3	4	2 to 5	2	2 to 3
	All	3	2 to 4	2	2 to 3
		**30% most**	**50% least**	**30% most**	**50% least**
Deprivation (% from areas in most deprived 30% and least deprived 50%)	1	-	-	-	-
	2	39.3	46.4	40.0	46.7
	3	41.4	34.5	50.0	21.4
	All	40.4	40.4	44.8	34.5
		**Male**	**Female**	**Male**	**Female**
Sex (% of each sex receiving the treatment by setting)	1	-	-	-	-
	2	72.7	64.7	27.7	35.5
	3	64.0	68.4	36.0	31.6
	All	69.0	66.7	31.0	33.3
