# Supplementary material for: COVID-19 managed on respiratory wards and intensive care units: Results from the national COVID-19 outcome report in Wales from March 2020 to December 2021
Source: PLoS One. 2024 Jan 19;19(1):e0294895. doi: 10.1371/journal.pone.0294895 (PMC10798461; doi:10.1371/journal.pone.0294895)
Supplement: S11 Table — (PDF) [file pone.0294895.s014.pdf]

S15 Table. Subgroup counts and percents: HFNO only

		Ward admissions with HFNO only			ICU admissions with HFNO only		
		Wave 1	Wave 2	Wave 3	Wave 1	Wave 2	Wave 3
		n (%)	n (%)	n (%)	n (%)	n (%)	n (%)
Age	18-39	- -	2 (5.7)	1 (3.4)	- -	2 (13.3)	2 (13.3)
	40-49	- -	1 (2.9)	2 (6.9)	- -	3 (20.0)	2 (13.3)
	50-59	- -	8 (22.9)	2 (6.9)	- -	4 (26.7)	4 (26.7)
	60-69	- -	10 (28.6)	9 (31.0)	- -	3 (20.0)	5 (33.3)
	70-79	- -	9 (25.7)	7 (24.1)	- -	3 (20.0)	2 (13.3)
	80+	- -	5 (14.3)	8 (27.6)	- -	0 (0.0)	0 (0.0)
	All	- -	35 (100)	29 (100)	- -	15 (100)	15 (100)
Sex	Male	- -	24 (68.6)	16 (55.2)	- -	9 (60.0)	9 (60.0)
	Female	- -	11 (31.4)	13 (44.8)	- -	6 (40.0)	6 (40.0)
	All	- -	35 (100)	29 (100)	- -	15 (100)	15 (100)
Comorbidities	0	- -	5 (14.3)	1 (3.4)	- -	0 (0.0)	2 (13.3)
	1	- -	5 (14.3)	3 (10.3)	- -	4 (26.7)	1 (6.7)
	2	- -	6 (17.1)	5 (17.2)	- -	3 (20.0)	6 (40.0)
	3	- -	12 (34.3)	5 (17.2)	- -	4 (26.7)	3 (20.0)
	4	- -	2 (5.7)	7 (24.1)	- -	1 (6.7)	2 (13.3)
	5+	- -	5 (14.3)	8 (27.6)	- -	3 (20.0)	1 (6.7)
	All	- -	35 (100)	29 (100)	- -	15 (100)	15 (100)
Deprivation	most 10%	- -	1 (3.6)	4 (13.8)	- -	0 (0.0)	2 (14.3)
	most 10-20%	- -	4 (14.3)	3 (10.3)	- -	4 (26.7)	2 (14.3)
	most 20-30%	- -	6 (21.4)	5 (17.2)	- -	2 (13.3)	3 (21.4)
	most 30-50%	- -	4 (14.3)	7 (24.1)	- -	2 (13.3)	4 (28.6)
	least 50%	- -	13 (46.4)	10 (34.5)	- -	7 (46.7)	3 (21.4)
	All	- -	28 (100)	29 (100)	- -	15 (100)	14 (100)
