# Supplementary material for: COVID-19 managed on respiratory wards and intensive care units: Results from the national COVID-19 outcome report in Wales from March 2020 to December 2021
Source: PLoS One. 2024 Jan 19;19(1):e0294895. doi: 10.1371/journal.pone.0294895 (PMC10798461; doi:10.1371/journal.pone.0294895)
Supplement: S12 Table — (PDF) [file pone.0294895.s015.pdf]

## S16 Table. Subgroup counts and percents: CPAP and invasive ventilation

| | | Ward admissions with CPAP | | | ICU admissions with CPAP | | | ICU admissions invasively ventilated | | |
|---|---|---|---|---|---|---|---|---|---|---|
| | | Wave 1 | Wave 2 | Wave 3 | Wave 1 | Wave 2 | Wave 3 | Wave 1 | Wave 2 | Wave 3 |
| | | n (%) | n (%) | n (%) | n (%) | n (%) | n (%) | n (%) | n (%) | n (%) |
| Age | 18-39 | 1 (1.6) | 5 (3.3) | 6 (7.6) | 3 (21.4) | 8 (10.7) | 3 (7.3) | 6 (6.1) | 5 (5.0) | 2 (4.5) |
| | 40-49 | 5 (7.9) | 11 (7.2) | 10 (12.7) | 3 (21.4) | 7 (9.3) | 7 (17.1) | 12 (12.1) | 17 (17.0) | 10 (22.7) |
| | 50-59 | 9 (14.3) | 28 (18.4) | 9 (11.4) | 2 (14.3) | 24 (32.0) | 15 (36.6) | 25 (25.3) | 27 (27.0) | 11 (25.0) |
| | 60-69 | 13 (20.6) | 34 (22.4) | 25 (31.6) | 6 (42.9) | 25 (33.3) | 7 (17.1) | 30 (30.3) | 32 (32.0) | 15 (34.1) |
| | 70-79 | 23 (36.5) | 43 (28.3) | 18 (22.8) | 0 (0.0) | 9 (12.0) | 6 (14.6) | 26 (26.3) | 19 (19.0) | 5 (11.4) |
| | 80+ | 12 (19.0) | 31 (20.4) | 11 (13.9) | 0 (0.0) | 2 (2.7) | 3 (7.3) | 0 (0.0) | 0 (0.0) | 1 (2.3) |
| | All | 63 (100) | 152 (100) | 79 (100) | 14 (100) | 75 (100) | 41 (100) | 99 (100) | 100 (100) | 44 (100) |
| Sex | Male | 42 (66.7) | 86 (56.6) | 47 (59.5) | 8 (57.1) | 44 (58.7) | 27 (65.9) | 69 (69.7) | 77 (77.0) | 28 (63.6) |
| | Female | 21 (33.3) | 66 (43.4) | 32 (40.5) | 6 (42.9) | 31 (41.3) | 14 (34.1) | 30 (30.3) | 23 (23.0) | 16 (36.4) |
| | All | 63 (100) | 152 (100) | 79 (100) | 14 (100) | 75 (100) | 41 (100) | 99 (100) | 100 (100) | 44 (100) |
| Comorbidities | 0 | 1 (1.6) | 14 (9.2) | 2 (2.5) | 6 (42.9) | 5 (6.7) | 7 (17.1) | 14 (14.1) | 17 (17.0) | 2 (4.5) |
| | 1 | 14 (22.2) | 21 (13.8) | 17 (21.5) | 2 (14.3) | 20 (26.7) | 9 (22.0) | 26 (26.3) | 26 (26.0) | 2 (4.5) |
| | 2 | 21 (33.3) | 38 (25.0) | 13 (16.5) | 1 (7.1) | 17 (22.7) | 6 (14.6) | 26 (26.3) | 18 (18.0) | 16 (36.4) |
| | 3 | 15 (23.8) | 25 (16.4) | 13 (16.5) | 3 (21.4) | 9 (12.0) | 11 (26.8) | 19 (19.2) | 19 (19.0) | 10 (22.7) |
| | 4 | 5 (7.9) | 24 (15.8) | 9 (11.4) | 0 (0.0) | 6 (8.0) | 6 (14.6) | 8 (8.1) | 8 (8.0) | 5 (11.4) |
| | 5+ | 7 (11.1) | 30 (19.7) | 25 (31.6) | 2 (14.3) | 18 (24.0) | 2 (4.9) | 6 (6.1) | 12 (12.0) | 9 (20.5) |
| | All | 63 (100) | 152 (100) | 79 (100) | 14 (100) | 75 (100) | 41 (100) | 99 (100) | 100 (100) | 44 (100) |
| Deprivation | most 10% | 12 (19.7) | 18 (12.3) | 7 (9.0) | 4 (36.4) | 4 (5.5) | 5 (12.8) | 13 (14.3) | 16 (17.8) | 4 (9.1) |
| | most 10-20% | 7 (11.5) | 15 (10.3) | 8 (10.3) | 0 (0.0) | 4 (5.5) | 4 (10.3) | 11 (12.1) | 10 (11.1) | 3 (6.8) |
| | most 20-30% | 8 (13.1) | 21 (14.4) | 10 (12.8) | 1 (9.1) | 14 (19.2) | 7 (17.9) | 15 (16.5) | 10 (11.1) | 12 (27.3) |
| | most 30-50% | 18 (29.5) | 27 (18.5) | 13 (16.7) | 2 (18.2) | 12 (16.4) | 7 (17.9) | 17 (18.7) | 25 (27.8) | 9 (20.5) |
| | least 50% | 16 (26.2) | 65 (44.5) | 40 (51.3) | 4 (36.4) | 39 (53.4) | 16 (41.0) | 35 (38.5) | 29 (32.2) | 16 (36.4) |
| | All | 61 (100) | 146 (100) | 78 (100) | 11 (100) | 73 (100) | 39 (100) | 91 (100) | 90 (100) | 44 (100) |
