# Supplementary material for: COVID-19 managed on respiratory wards and intensive care units: Results from the national COVID-19 outcome report in Wales from March 2020 to December 2021
Source: PLoS One. 2024 Jan 19;19(1):e0294895. doi: 10.1371/journal.pone.0294895 (PMC10798461; doi:10.1371/journal.pone.0294895)
Supplement: S13 Table — (PDF) [file pone.0294895.s016.pdf]

S17 Table. Subgroup summary statistics: invasive ventilation

	Wave	Invasively ventilated	
		Median	IQR
Age	1	62	53 to 70
	2	60	51.5 to 67.5
	3	58	48.5 to 67
	All	60	52 to 68
Comorbidities	1	2	1 to 3
	2	2	1 to 3
	3	3	2 to 4
	All	2	1 to 3
		**30% most**	**50% least**
Deprivation (% from areas in most deprived 30% and least deprived 50%)	1	42.9	38.5
	2	40.0	32.2
	3	43.2	36.4
	All	41.8	35.6
		**Male**	**Female**
Sex (%)	1	69.7	30.3
	2	77.0	23.0
	3	63.6	36.4
	All	71.6	28.4
