# Supplementary material for: COVID-19 managed on respiratory wards and intensive care units: Results from the national COVID-19 outcome report in Wales from March 2020 to December 2021
Source: PLoS One. 2024 Jan 19;19(1):e0294895. doi: 10.1371/journal.pone.0294895 (PMC10798461; doi:10.1371/journal.pone.0294895)
Supplement: S14 Table — (PDF) [file pone.0294895.s017.pdf]

S18 Table. CPAP subgroup crude outcomes

	Wave	Cases	Deaths			p value
		n	n	%	(95% CI)	
Ward admissions without any oxygen therapy	1	567	126	22.2	(19.0 to 25.8)	1 v 2: p<0.01
	2	760	107	14.1	(11.8 to 16.7)	2 v 3: p=0.48
	3	497	63	12.7	(10.0 to 15.9)	3 v 1: p<0.01
Ward admissions with oxygen therapy only	1	268	91	34.0	(28.5 to 39.8)	1 v 2: p<0.01
	2	518	84	16.2	(13.3 to 19.6)	2 v 3: p=0.21
	3	279	36	12.9	(9.5 to 17.3)	3 v 1: p<0.01
Ward admissions with HFNO only	1	-	-	-	-	-
	2	35	15	42.9	(28.0 to 59.1)	2 v 3: p=0.87
	3	29	13	44.8	(28.4 to 62.5)	-
ICU admissions with HFNO only	1	-	-	-	-	-
	2	15	5	33.3	(15.2 to 58.3)	2 v 3: p=0.41
	3	15	3	20.0	(7.0 to 45.2)	-
Ward admissions with CPAP only	1	63	40	63.5	(51.1 to 74.3)	1 v 2: p=0.03
	2	152	71	46.7	(39.0 to 54.6)	2 v 3: p=0.28
	3	79	31	39.2	(29.2 to 50.3)	3 v 1: p<0.01
ICU admissions with CPAP only	1	14	5	35.7	(16.3 to 61.2)	1 v 2: p=0.36
	2	75	18	24.0	(15.8 to 34.8)	2 v 3: p=0.80
	3	41	9	24.6	(12.0 to 36.7)	3 v 1: p=0.31
ICU admissions with invasive ventilation	1	99	61	61.6	(51.8 to 70.6)	1 v 2: p=0.82
	2	100	60	60.0	(50.2 to 69.1)	2 v 3: p=0.54
	3	44	24	54.6	(40.1 to 68.3)	3 v 1: p=0.43
