# Supplementary material for: COVID-19 managed on respiratory wards and intensive care units: Results from the national COVID-19 outcome report in Wales from March 2020 to December 2021
Source: PLoS One. 2024 Jan 19;19(1):e0294895. doi: 10.1371/journal.pone.0294895 (PMC10798461; doi:10.1371/journal.pone.0294895)
Supplement: S15 Table — (PDF) [file pone.0294895.s018.pdf]

S19 Table. Univariate logistic regression, CPAP subgroup

Variable		Coefficient (β)	SE	Wald χ^2	P value	Odds Ratio	95% CI
CPAP	CPAP in ICU	-1.051	0.235	-4.5	<0.01	0.35	0.22 to 0.55
	(Baseline) CPAP on Ward	0.000				1.00	
Wave	1	0.811	0.277	2.9	<0.01	2.25	1.31 to 3.87
	(Baseline) 2	0.000				1.00	
	3	-0.276	0.240	-1.2	<0.01	0.76	0.47 to 1.21
Comorbidities	0	-2.375	0.761	-3.1	<0.01	0.09	0.02 to 0.41
	1	-0.515	0.324	-1.6	0.11	0.60	0.32 to 1.13
	(Baseline) 2	0.000				1.00	
	3	-0.017	0.316	-0.1	0.96	0.98	0.53 to 1.83
	4	0.704	0.360	2.0	0.05	2.02	1.00 to 4.10
	5+	0.556	0.309	1.8	0.07	1.74	0.95 to 3.19
Age	18-39	-1.412	0.651	-2.2	0.03	0.24	0.07 to 0.87
	40-49	-1.766	0.563	-3.1	<0.01	0.17	0.06 to 0.52
	50-59	-1.516	0.392	-3.9	<0.01	0.22	0.10 to 0.47
	(Baseline) 60-69	0.000				1.00	
	70-79	1.179	0.295	4.0	<0.01	3.25	1.82 to 5.80
	80+	2.054	0.402	5.1	<0.01	7.80	3.55 to 17.13
Sex	Female	0.003	0.205	0.0	0.99	1.00	0.67 to 1.50
	(Baseline) Male	0.000				1.00	
Deprivation	Most 10%	-0.417	0.339	-1.2	0.22	0.66	0.34 to 1.28
	Most 10-20%	-0.091	0.365	-0.2	0.80	0.91	0.45 to 1.87
	Most 20-30%	0.304	0.297	1.0	0.31	1.35	0.76 to 2.43
	Most 30-50%	0.004	0.274	0.0	0.99	1.00	0.59 to 1.72
	(Baseline) Least 50%	0.000				1.00	
