# Supplementary material for: COVID-19 managed on respiratory wards and intensive care units: Results from the national COVID-19 outcome report in Wales from March 2020 to December 2021
Source: PLoS One. 2024 Jan 19;19(1):e0294895. doi: 10.1371/journal.pone.0294895 (PMC10798461; doi:10.1371/journal.pone.0294895)
Supplement: S16 Table — (PDF) [file pone.0294895.s019.pdf]

S21 Table. Multivariable logistic regression, CPAP subgroup

Variable		Coefficient (β)	SE	Wald χ^2	P value	Odds Ratio	95% CI
CPAP	CPAP in ICU	-0.121	0.301	-0.4	0.69	0.89	0.49 to 1.60
	(Baseline) CPAP on Ward	0.000				1.00	
Wave	1	1.134	0.364	3.1	<0.01	3.11	1.52 to 6.34
	(Baseline) 2	0.000				1.00	
	3	-0.180	0.297	-0.6	0.54	0.84	0.47 to 1.49
Comorbidities	0	-1.811	0.838	-2.2	0.03	0.16	0.03 to 0.84
	1	-0.346	0.397	-0.9	0.38	0.71	0.33 to 1.54
	(Baseline) 2	0.000				1.00	
	3	-0.249	0.387	-0.6	0.52	0.78	0.37 to 1.66
	4	0.688	0.435	1.6	0.11	1.99	0.85 to 4.66
	5+	0.535	0.381	1.4	0.16	1.71	0.81 to 3.61
Age	18-39	-1.322	0.701	-1.9	0.06	0.27	0.07 to 1.05
	40-49	-1.631	0.578	-2.8	<0.01	0.20	0.06 to 0.61
	50-59	-1.290	0.415	-3.1	<0.01	0.28	0.12 to 0.62
	(Baseline) 60-69	0.000				1.00	
	70-79	1.108	0.319	3.5	<0.01	3.03	1.62 to 5.66
	80+	1.944	0.424	4.6	<0.01	6.99	3.04 to 16.04
Sex	Female	-0.144	0.260	-0.6	0.58	0.87	0.52 to 1.44
	(Baseline) Male	0.000				1.00	
Deprivation	Most 10%	-0.437	0.432	-1.0	0.31	0.65	0.28 to 1.51
	Most 10-20%	0.121	0.461	0.3	0.79	1.13	0.46 to 2.79
	Most 20-30%	0.419	0.383	1.1	0.27	1.52	0.72 to 3.22
	Most 30-50%	-0.147	0.353	-0.4	0.68	0.86	0.43 to 1.72
	(Baseline) Least 50%	0.000				1.00	
	Constant	-0.567	0.379				
