# Supplementary material for: COVID-19 managed on respiratory wards and intensive care units: Results from the national COVID-19 outcome report in Wales from March 2020 to December 2021
Source: PLoS One. 2024 Jan 19;19(1):e0294895. doi: 10.1371/journal.pone.0294895 (PMC10798461; doi:10.1371/journal.pone.0294895)

S10 Appendix. Goodness of fit: whole cohort model

There were 5,611 observations with 1,329 covariate patterns in this dataset. The Pearson chi-square goodness of fit test indicated no problems with the fit of the model (p=0.25).

The area under the Receiver Operating Characteristic (ROC) curve suggested the model had satisfactory discriminatory powers (0.77).

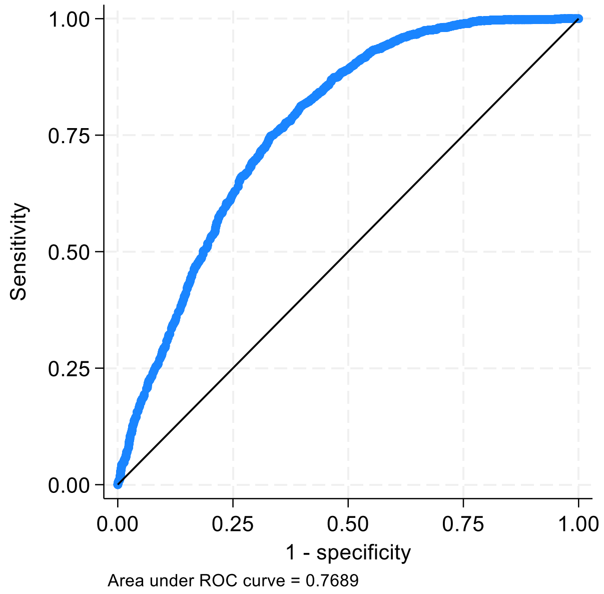

Plots of the predicted values showed some degree of separation between the outcome groups in terms of estimated probabilities from the model, though both groups were largely below 0.5.

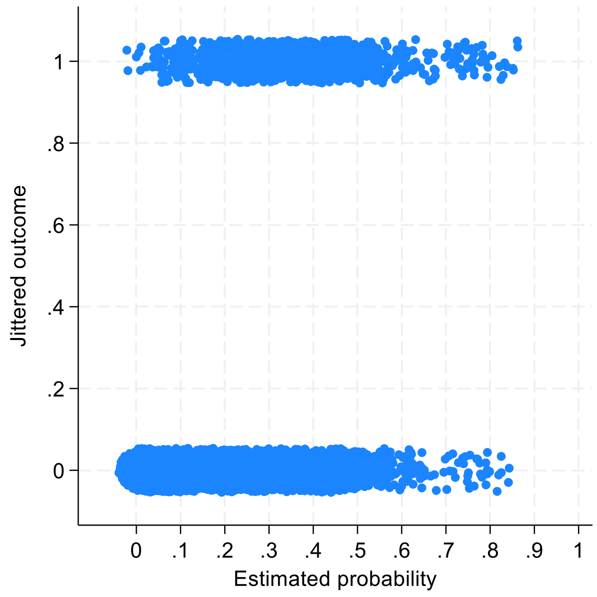

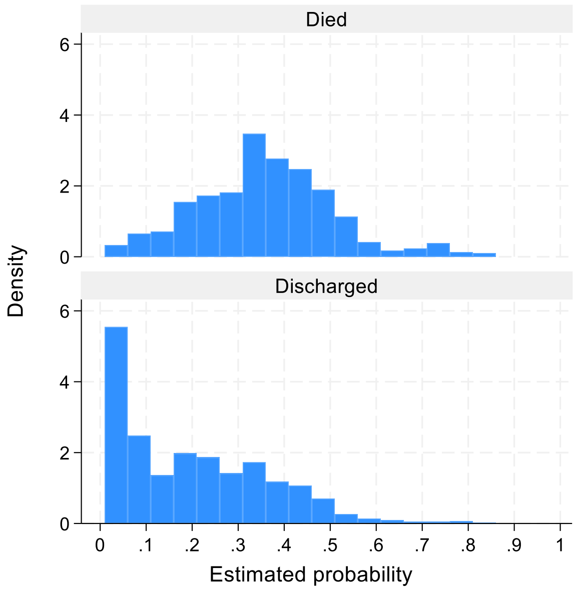

Supplement: S1 Appendix — (PDF) [file pone.0294895.s020.pdf]
