# Supplementary material for: COVID-19 managed on respiratory wards and intensive care units: Results from the national COVID-19 outcome report in Wales from March 2020 to December 2021
Source: PLoS One. 2024 Jan 19;19(1):e0294895. doi: 10.1371/journal.pone.0294895 (PMC10798461; doi:10.1371/journal.pone.0294895)
Supplement: S2 Appendix — (PDF) [file pone.0294895.s021.pdf]

S11 Appendix. Regression diagnostics: whole cohort model

Inspection of plots of the estimated probabilities against leverage, change in Pearson chi-square, change in deviance and Cook's distance indicated a number of covariate patterns that may have been poorly fitted by the model.

The most extreme values towards the upper corners were investigated along with those that seemed some distance from the general patterns. The most extreme values for change in Chi square were for covariate patterns of younger people on the ward, generally female, who had low estimated probabilities of death but died. The higher points more centrally located were typically covariate patterns of older males with high comorbidities but with a relatively high proportion of discharges. One particular such covariate pattern is seen in the most extreme Cook's distance point, consisting of 19 males with 5+ comorbidities. None of the covariate patterns investigated seemed clinically implausible and all records were retained in the model.

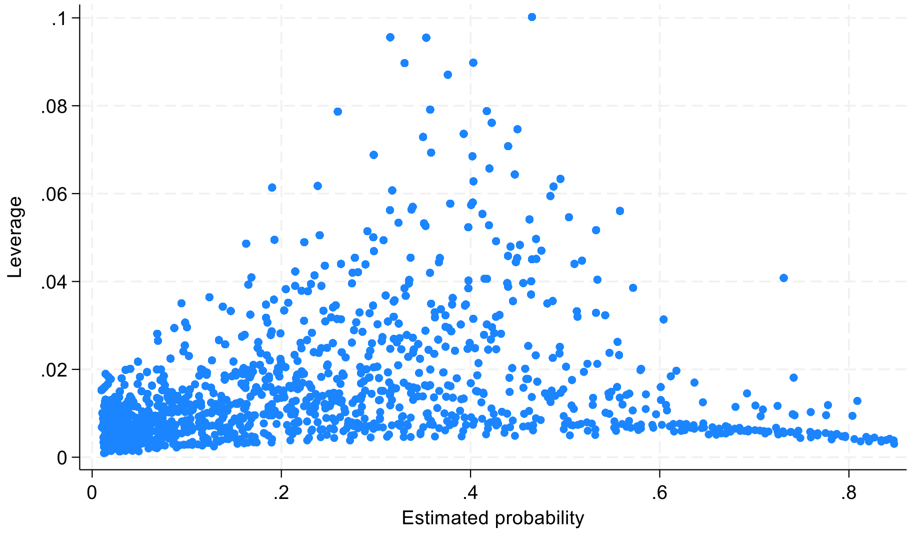

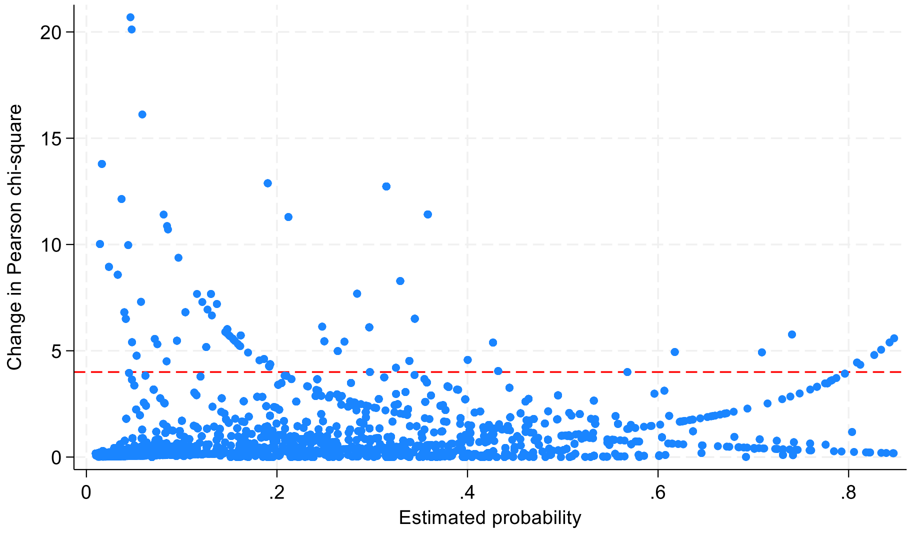

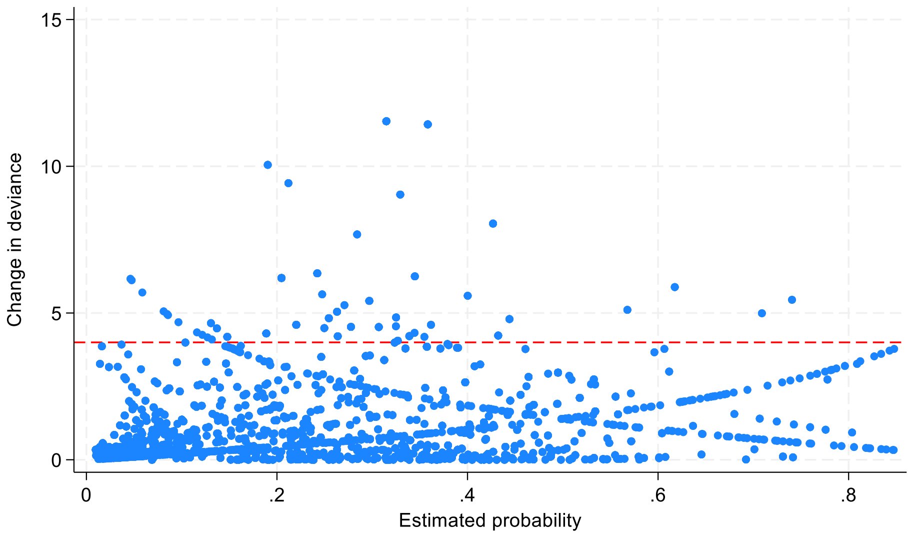

Change in deviance
Estimated probability

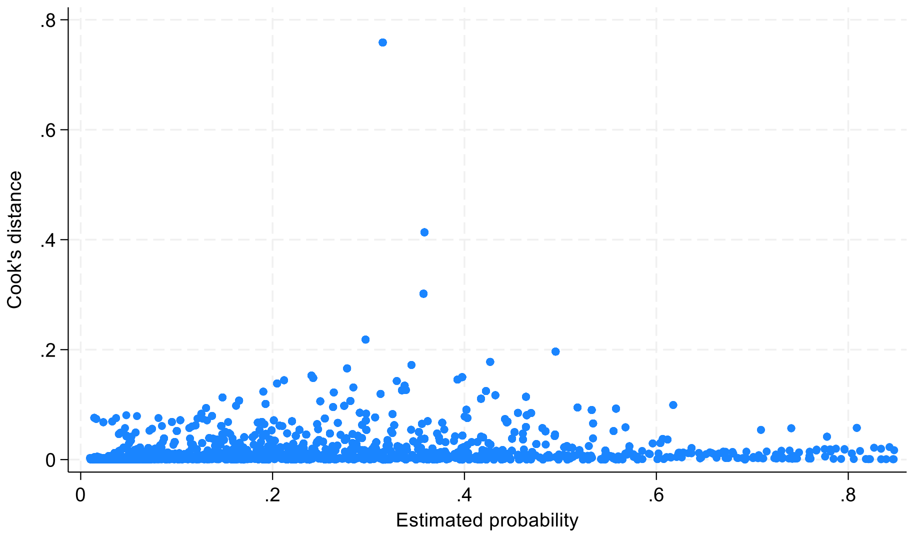

Cook's distance
Estimated probability

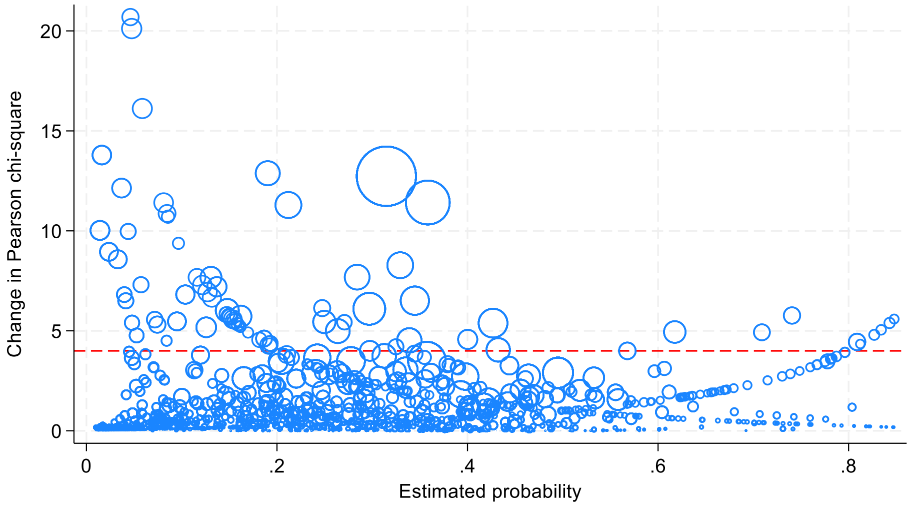

Change in Pearson chi-square
Estimated probability
circle size weighted by Cook's distance
