# Supplementary material for: COVID-19 managed on respiratory wards and intensive care units: Results from the national COVID-19 outcome report in Wales from March 2020 to December 2021
Source: PLoS One. 2024 Jan 19;19(1):e0294895. doi: 10.1371/journal.pone.0294895 (PMC10798461; doi:10.1371/journal.pone.0294895)

S22 Appendix. Goodness of fit: CPAP subgroup model

There were 408 observations with 321 covariate patterns in this dataset. The Pearson chi-square goodness of fit test indicated no problems with the fit of the model (p=0.30). As a precaution due to the somewhat closeness of the observations and the covariate patterns, the Hosmer-Lemeshow test was also conducted and also indicated not problems with the fit of the model (p=0.70).

The area under the Receiver Operating Characteristic (ROC) curve suggested the model had excellent discriminatory powers (0.84).

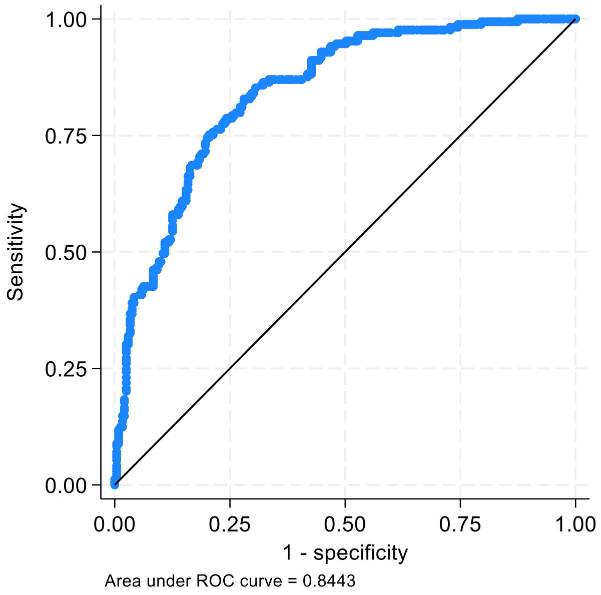

Plots of the predicted values showed fairly clear separation of the outcome groups in terms of estimated probabilities from the model.

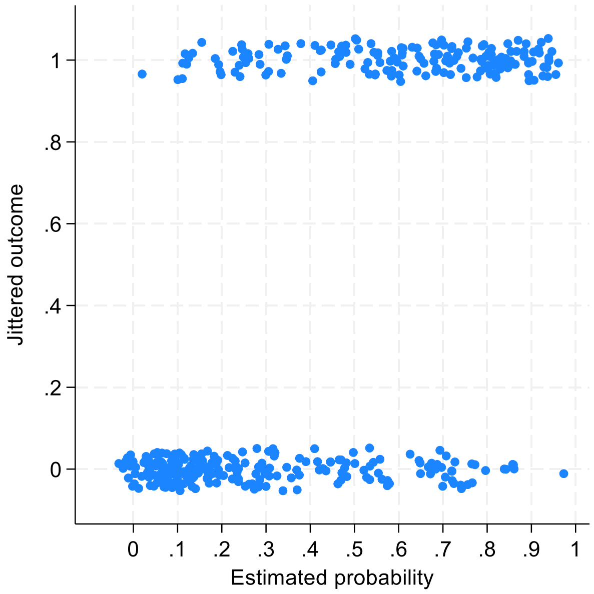

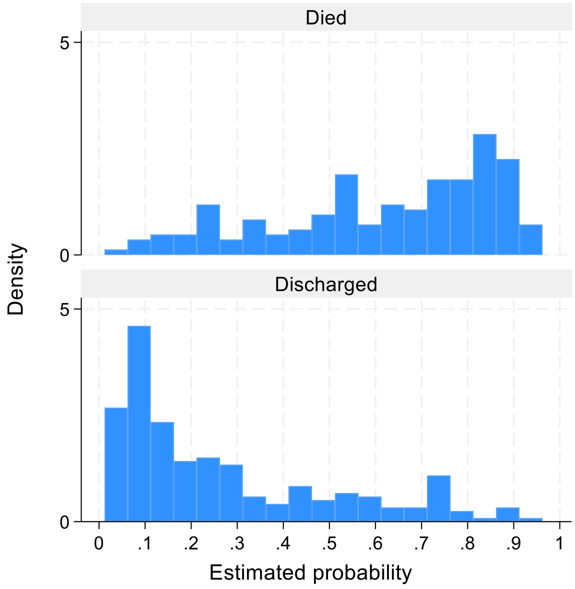

Supplement: S3 Appendix — (PDF) [file pone.0294895.s022.pdf]
