# Supplementary material for: COVID-19 managed on respiratory wards and intensive care units: Results from the national COVID-19 outcome report in Wales from March 2020 to December 2021
Source: PLoS One. 2024 Jan 19;19(1):e0294895. doi: 10.1371/journal.pone.0294895 (PMC10798461; doi:10.1371/journal.pone.0294895)
Supplement: S4 Appendix — (PDF) [file pone.0294895.s023.pdf]

S23 Appendix. Regression diagnostics: CPAP subgroup model

Inspection of plots of the estimated probabilities against leverage, change in Pearson chi-square, change in deviance and Cook's distance indicated a number of covariate patterns that may have been poorly fitted by the model.

The most extreme values towards the upper corners were investigated along with those that seemed some distance from the general patterns. They could be clearly separated into two groups: younger patients who received CPAP in ICU (so had low estimated probabilities of death) but died, and older patients who received CPAP on the ward (so had high estimated probabilities of death) but were discharged. The particularly notable covariate pattern on all the charts with an estimated probability of 0.2 and lying a long way from the other points consisted of two males aged 60-69 with no comorbidities who received CPAP in ICU but died. The most extreme point for change in chi-square and change in deviance with low estimated probability consisted of one female aged 50-59 with one comorbidity who received CPAP in ICU but died. None of the covariate patterns investigated seemed clinically implausible and all records were retained in the model.

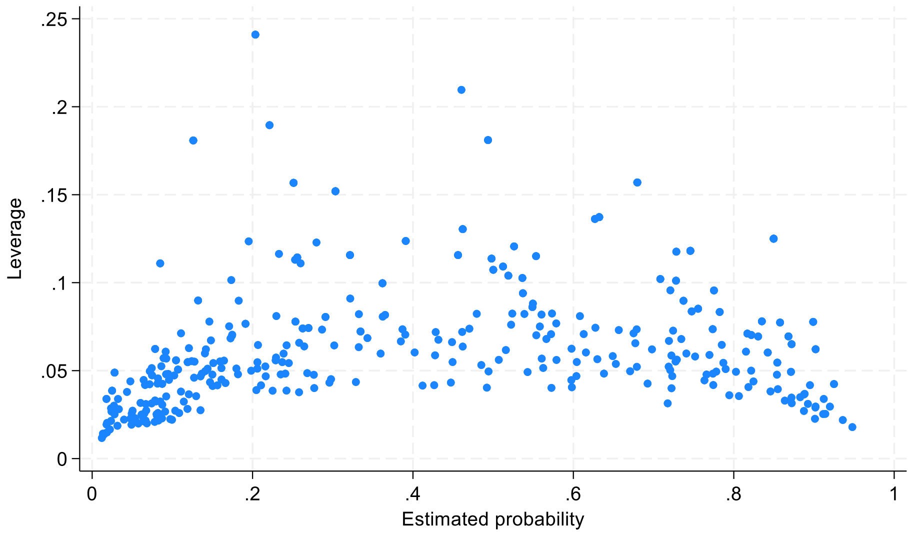

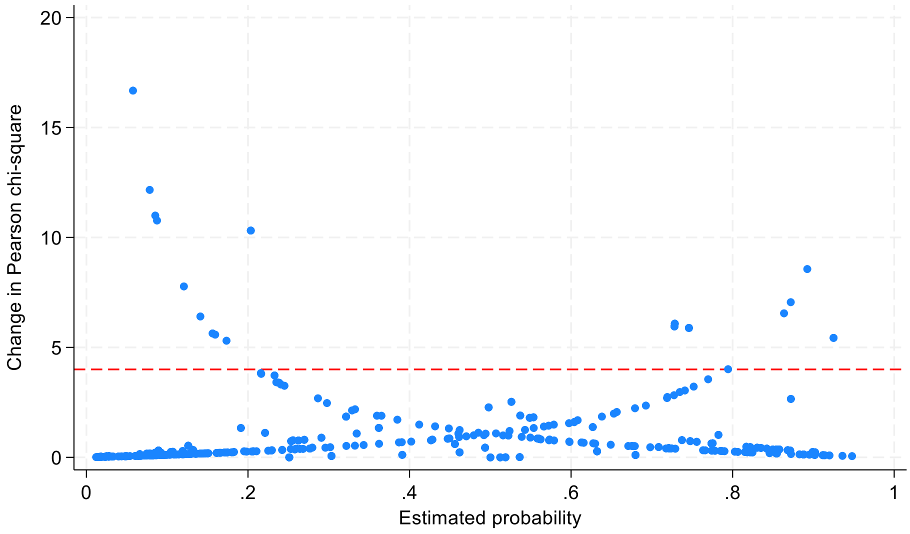

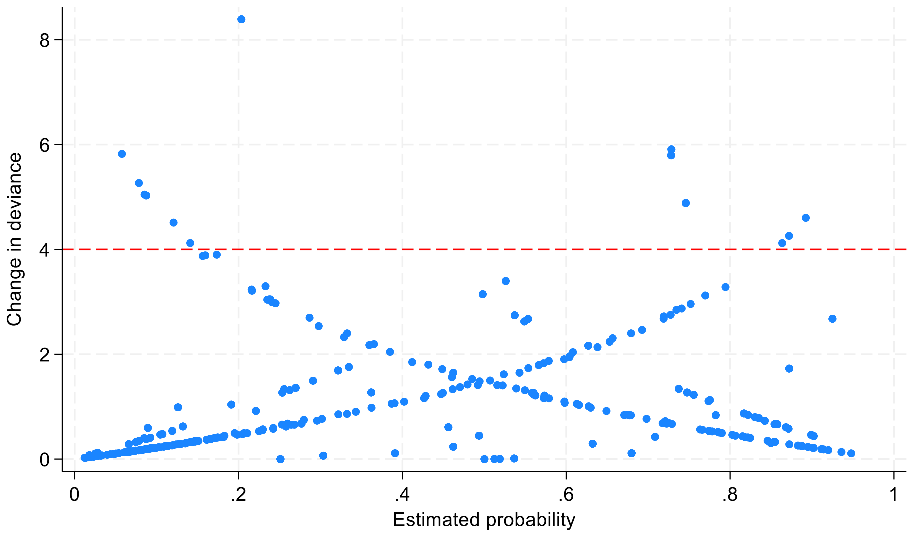

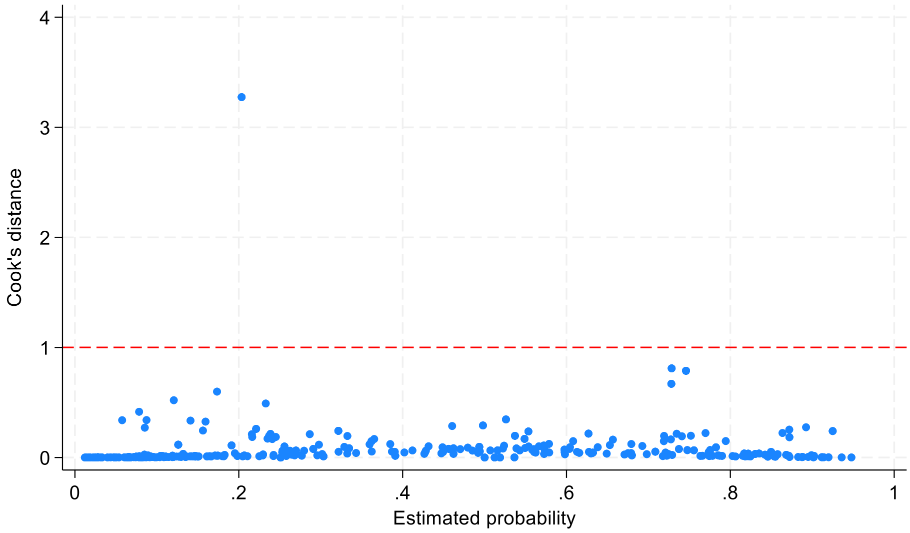

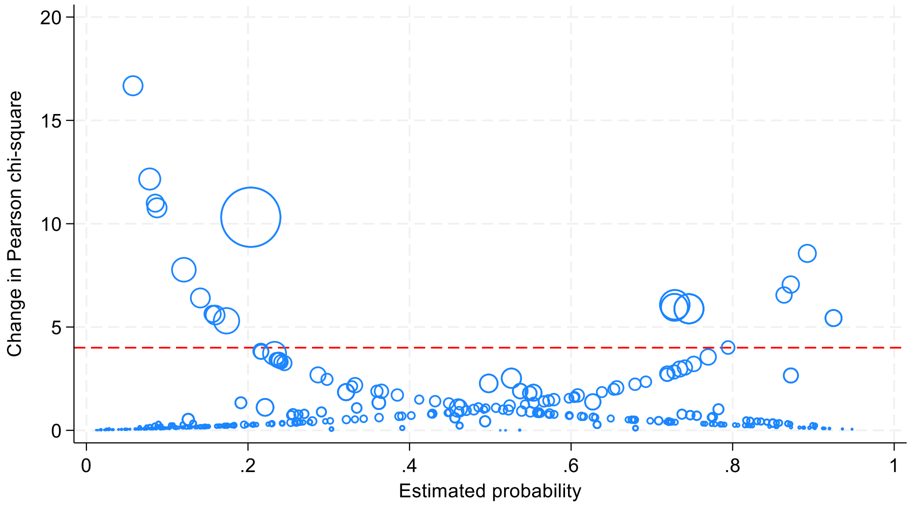

circle size weighted by Cook's distance
