# Supplementary material for: COVID-19 managed on respiratory wards and intensive care units: Results from the national COVID-19 outcome report in Wales from March 2020 to December 2021
Source: PLoS One. 2024 Jan 19;19(1):e0294895. doi: 10.1371/journal.pone.0294895 (PMC10798461; doi:10.1371/journal.pone.0294895)
Supplement: Attachment — Submitted filename: PLOSONE ReviewComments_20230513.docx [file pone.0294895.s024.docx]

| **Journal requirements** | | **Response** |
| --- | --- | --- |
| JR1 | Please ensure that your manuscript meets PLOS ONE's style requirements, including those for file naming. |  |
| JR2 | We note that the grant information you provided in the ‘Funding Information’ and ‘Financial Disclosure’ sections do not match.  When you resubmit, please ensure that you provide the correct grant numbers for the awards you received for your study in the ‘Funding Information’ section. | Have written no specific funding for this study on MS and online |
| JR3 | Thank you for stating the following financial disclosure:  "Welsh Government Funds the Respiratory Health Implementation Group (RHIG) and Respiratory Innovation Wales. RHIG fund the Institute for Clinical Science and Technology (ICST), who create and implement the digital innovations."  Please state what role the funders took in the study.  If the funders had no role, please state: "The funders had no role in study design, data collection and analysis, decision to publish, or preparation of the manuscript." | The funders had no role in the study design, data collection and analysis, decision to publish or preparation of the manuscript |
| JR4 | Thank you for stating the following in your Competing Interests section:  “no authors have competing interests”  Please complete your Competing Interests on the online submission form to state any Competing Interests. If you have no competing interests, please state "The authors have declared that no competing interests exist.", as detailed online in our guide for authors at [http://journals.plos.org/plosone/s/submit-now](http://scanmail.trustwave.com/?c=261&d=wNTb5Boroe-Et8blfvKtoZRAFhBLa_LNvg2IbADcjg&u=http%3a%2f%2fjournals%2eplos%2eorg%2fplosone%2fs%2fsubmit-now).   This information should be included in your cover letter; we will change the online submission form on your behalf. | The authors have declared that no competing interests exist |
| JR5 | Please include captions for your Supporting Information files at the end of your manuscript, and update any in-text citations to match accordingly. Please see our Supporting Information guidelines for more information |  |
|  | | |
| **Reviewer 1** | | **Response** |
| 1.1 | Introduction / LL43-50: The authors make a huge point of the national guideline. This part is quite unexpected and please justify why this part is required, as it does not seem to be relevant to the data presented. Or was the guideline and database tighly connected? Please clarify. | We do make a strong claim here. We believe that this is the first time that a guideline which rapidly responded to changes in information was implemented at a national level, and we actually measured user engagement and reached 100% of the target audience. This is important because we have to an extent standardised care, particularly promoting ward management of COVID-19 pneumonitis with oxygen and CPAP/HFNO. ICNARC data demonstrate that far more CPAP was done in ICU in England than in Wales which supports the fact that our guideline was widely adopted. The database was created by the same organisation (Institute for clinical science and technology) as who created the guideline and utilised the same implementation principles. |
| 1.2 | Introduction / L50: The last meaning on avoiding invasive mechanical ventilation whenever possible spikes an interest. This strategy is similar to what was experienced from northern Italy. In light of your results, I think your viewpoint regarding this practice would be quite interesting. Did the strategy change over time when pressure on ICU/ventilator beds decreased? | No, we continued to manage COVID-19 pneumonitis predominantly on respiratory wards. Interestingly, it was wave 1, when there was the least pressure on beds (because hospitals had been emptied of patients), whereas waves 2 and 3 had sustained bed pressures. There continued to be great debate amongst the ICU community about the optimum time to intubate. In general, those patients who deteriorated after >1 week of CPAP/HFNO on wards did not do well on ICU. There were mortality improvements with new treatments and vaccination as we have shown |
| 1.3 | Method / L967: Please specify when mortality was assessed. During index admission? Hospital mortality? 30d? If patients were discharged to for example a nursing facility and succumbed shortly thereafter, was this registered as an primary event outcome? Please specify. | This was only during the index admission. Mortality was a mandatory field. We did not measure mortality after discharge. However, care homes did not accept patients back until they had completely recovered from COVID and repeatedly tested negative. We agree that this is an important point, and have added this to the limitations section |
| 1.4 | Method / Statistical analysis: Appears to be sound. | Thank you, multiple changes to new MS |
| 1.5 | Results / Well presented, Fig 1 and Fig 2 are informative and yields relevant information. | Thank you, has been changed according to reviewer 3 |
| 1.6 | Results / Just out of curiosity, why did you chose the specific baseline/reference categories? Eg Wave 2 as reference instead of 1 or 3? (No need to revise!) | When the number of observations varies greatly between levels the most robust results (smallest standard errors) will be obtained by choosing the largest level as the baseline. This guided our choice of baseline/reference categories for these analyses. |
| 1.7 | Discussion / LL216-218: Consider omitting this, the aim was already stated. I would rather see your major findings first in the discussion. | Removed as suggested |
| 1.8 | Discussion / LL227-228: I would add that the reduction in mortality over was likely due to.... There is no data to support increasing experience - for pharmaceuticals, pls refer to the supplement table. | Changed as suggested |
| 1.9 | Discussion / LL230-233: Does your data support this? | No, our data does not. However, It was clear that experience was gained and teams got better in managing COVID pneumonitis. Getting a CTPA to exclude PE became standard and utilising PCT to guide concurrent antibiotic management was also widely used for example. I think it would be wrong not to consider this, although we haven’t formally measured it. Have added the reference about pneumomediastinum. |
| 1.10 | Discussion / LL236-240: I think this is a v imp finding - consider adding that this could be attributed to the publicly funded healthcare system (if you agree of course!) | We do agree it is important. However, we do not want to overstate the case, and recognise that there were other factors that influenced mortality related to deprivation beyond what we have measured |
| 1.11 | Discussion / LL255-265: Here you pick up on the topic I commented on earlier - I would like to see a deeper analysis. In hindsight, would you do the same choice again? This is important for future desicion-making! Did the propotion of patients on invasive ventilation change over time? |  |
| 1.12 | Discussion / LL282: Consider adding that less than half the hospitalised patients were registered to the database. | Stated at beginning of discussion- 40% |
| 1.13 | I enjoyed reading this report and I think it is valuable for future decision-making and hoping that my feedback could help improving the report further. | Thank you, we are grateful for your time and helpful comments. |
|  | | |
| **Reviewer 2** | | **Response** |
| 2.1 | Data are plural and the text should be updated accordingly (e.g. line 20 “data were collected” rather than “data was collected”) | changed |
| 2.2 | Abstract, methods – line 23, please define adults (> 18 years). | Done line 26 |
| 2.3 | Abstract, methods – suggest including a sentence regarding the statistical analyses performed. | Multivariable logistic regression models assessed variable effects on mortality. Added to methods in abstract |
| 2.4 | Abstract, results – suggest including the number of hospitals | Agree, changed line 19 |
| 2.5 | Ensure that “COVID-19” is used throughout the text rather than “COVID” (e.g. lines 50, 58 etc.) | Agree, changed |
| 2.6 | Methods, line 65 – suggest including additional information to describe the acute care hospitals if available (e.g. average bed size, adult vs. pediatric sites, teaching vs. non-teaching hospitals) | Have included median number of beds and IQR lines 76-77 |
| 2.7 | Methods, line 69 – please spell out HB the first time it is referenced | done |
| 2.8 | Methods, line 76 “continuous notes review by local clinical teams” – suggest specifying or describing what this means. Does it mean that admissions records were checked to see which ones had clinician notes available? Please clarify. | No, it means that local teams checked that the patient records identified from the Public Health Wales dataset fulfilled the criteria that we set out (LOS>1 day, age over 18 etc). This sentence is removed as we detail removal of duplicates, LOS <1 Day etc in results |
| 2.9 | Methods – suggest spelling out CPAP and defining HFNO which is referenced in the results but not in the methods | CPAP defined line 71 |
| 2.10 | Methods, lines 85-87 – suggest providing additional details regarding WIMD, specifically defining the levels or categories of deprivation status (e.g. 50% most deprived). | The Welsh Index of Multiple Deprivation (WIMD) is a small area measure of relative deprivation. It was developed as the official deprivation measure by the Welsh Government whose preferred use is in five groups: most deprived 10%; most deprived 10-20%, most deprived 20-30%, most deprived 30-50%, least deprived 50%. These groups are considered to be most alike internally and sufficiently distinct from each other. In our analysis due to small counts we combined the three most deprived groups to make a most deprived 30% group.  Wording in paper changed to:  We used the Welsh Governments preferred grouping of WIMD, though for some analyses collapsed the three most deprived groups into a single group of the 30% most deprived due to small counts in those smaller groups. (lines 95-98)  Ref for the groups:  [Welsh Index of Multiple Deprivation (WIMD): A guide to analysing indicator data, 2019 onwards (gov.wales)](https://www.gov.wales/sites/default/files/statistics-and-research/2023-01/welsh-index-of-multiple-deprivation-wimd-a-guide-to-analysing-indicator-data-2019-onwards.pdf) |
| 2.11 | Methods, line 96 – please define mortality. It is all-cause in-hospital mortality? 30-day mortality? | Changed to in hospital mortality line 112 |
| 2.12 | Methods, lines 102-103 – if possible, suggest indicating why some post codes did not link to a deprivation status (e.g. outside of geographic area of study? Or not mapped to status?) | That is correct. Some post codes did not link to deprivation status or were outside Wales. Added line 120-1 |
| 2.13 | Methods, line 105 – if possible, please provide further information regarding how missing data imputation was used. | No missing data imputation was used, the submitted manuscript had lost this critical first word off the sentence in question (line 122). Incomplete records were excluded from the modelling exercise. |
| 2.14 | Methods, line 105-106 – to confirm was BMI excluded from the analysis due to incomplete records? Please clarify | Yes that is correct. Poorly recorded in notes |
| 2.15 | Methods, line 109 – Health Boards is spelled out here. For consistency, suggest spelling out the first time it is used than consistently using HB. | changed |
| 2.16 | Results, line 133 – lower case “wave”, for consistency suggest using capital “Wave”. | changed |
| 2.17 | Results, lines 133-135 – the term patients, datasets and patient dataset are used interchangeably here. Suggest selecting one term to use consistently. | records used lines 156-158 |
| 2.18 | Results, lines 138-139 – is this referencing the median or average age? Please clarify. And “those admitted in Wave 3 were 4 years younger” – the median or average age was 4 years younger? | Median age added to text line 160 |
| 2.19 | Results, lines 139-140 “likely reflecting increased vaccination rates in the older age groups protecting against admission” – this is not a result but better suited in the discussion. Also, are there data to support this statement? Are population level age-stratified vaccination rates available? | Removed from results |
| 2.20 | Table 1 – consider adding p value results. | Table 1 changed as requested by review 3 |
| 2.21 | Results, lines 152-159 – suggest to include key relevant data (e.g. odds ratios and 95% confidence intervals) either in the text or figures. | Figure 2 OR and 95% CI |
| 2.22 | Results, lines 167-175 – as above, helpful to have odds ratios and 95% CI reported here for key results. |  |
| 2.23 | Results, lines 167-175 – suggest reporting as “odds of death” (e.g. the effect of age persisted with a lower odds of death at lower age…”) |  |
| 2.24 | Results, lines 193-195 – suggest confirming that this difference is statistically significant? Regardless, is it clinically significant? A difference of 1 comorbidity? | The difference (p<0.01) is statistically significant, but more importantly the odds ratios from the modelling suggest a significant difference between 2 and 3 comorbidities in terms of death. |
| 2.25 | Results, lines 208-209 – suggest adding definition of fully vaccinated in methods. Also, suggest clarifying sentence to “The overall mortality among unvaccinated patients was 20.5%, compared to 4.8% among patients who were fully vaccinated.” | Changed in methods (line 95)  And changed in results (line 180-1) |
| 2.26 | Results, lines 211-212 – suggest comparing to the same group (e.g. 81% were unvaccinated compared to 26.6% unvaccinated). The comparison of unvaccinated to vaccinated is not useful. |  |
| 2.27 | Discussion, line 219 – the data represents approximated 40% of total CA COVID admissions in acute hospitals in Wales? Of do the data represent 40% of all admissions (COVID and non-COVID)? Perhaps clarify because above it is stated that every acute hospital in Wales is captured in these data. | Its 40% of all COVID admissions. Although its more likely to be closer to 45% because the denominator data included inaccuracies |
| 2.28 | Discussion, line 223 – suggested updating to “our patients had higher mortality associated with increasing…” | changed |
| 2.29 | Discussion, line 247 – suggest using consistent terminology “fully vaccinated” vs. “double vaccinated with a booster” | Changed to fully vaccinated line |
| 2.30 | Discussion, line 247 – hesitant to use the word “confirm” here as this study did not control for other factors which may have either contributed to death (e.g. underlying comorbidities, age etc.) or improved chance of survival (such as age or prior/natural immunity) | Changed to ‘support the strong..’ |
| 2.31 | Discussion, lines 247-248 – cannot conclude that vaccines protect against hospitalization as this was not measured in this study (you would need community level data to compare to those CA COVID-19 positive not hospitalized by vaccination status). | As above |
| 2.32 | Discussion, lines 266 – consider conducting a multivariable logistic regression to compare those who received CPAP and those who did not with the outcome of mortality to provide further support/strength this conclusion. | We have done this |
| 2.33 | Figure 1 – should the grey bar be “non ICU admissions”? | This figure has been changed as per reviewer 3 comments and now shows mortality by site of care and treatment modality and Wave (figure 3) |
|  | | |
| **Reviewer 3** | | **Response** |
| 3.1 | The attempt to standardise management across Welsh hospitals and collect data about this was laudable. There is a lot of potentially interesting data here but the presentation of the results is disorganised. The paper lacks a clear aim. The paper falls between a scientific paper aiming to address a specific research question and a generic health department style report covering multiple aspects of care provided to COVID patients. If the aim is the former, it should be structured to address a specific question (e.g. change in mortality over the 3 waves). If the aim is the latter, it should be structured as a report with more complete ordered information with less commentary, although this then might be difficult to meet the requirements for publication as a scientific paper, and would be better suited to an on-line health department report. | We thank this author for his incisive comments. We entirely agree that this goes to the heart of the main weakness of this paper.  Our aim is for this to be a scientific paper. We argue that the guideline implementation and standardisation with widespread adoption of ward based care means that the main aim of this paper is to describe the outcomes of oxygen, CPAP and HFNO in wards and compare it to that in ICU. A secondary aim is to describe the mortality changes over successive waves. |
| 3.2 | The authors have also focused in on a result from a subgroup (CPAP in ICU) which does not appear to be a primary aim of the paper. If the main aim is to determine the how outcomes differ between patients who are treated with CPAP in the ward as opposed to ICU, then there should be a much more robust analysis to back up their findings, and this should become the focus of the paper. (See later comment also). | We have removed HFNO from this group and compared the CPAP on wards and ICU on univariate and multivariate analysis. |
| 3.3 | Outcomes from respiratory wards and ICUs are reported. Does this mean that patients admitted to other wards are not included? What happened to COVID patients put on other wards or have all patients admitted with COVID been included in the study? | All patients admitted with COVID-19 fulfilling our definition of community acquired were included. Those with any level of oxygen requirement were generally managed on respiratory wards, those with no oxygen requirement were managed on general wards. All were included |
| 3.4 | Do the authors have information on how many patients received invasive ventilation? This is vital to interpret the prevalence and outcomes associated with provision of other respiratory / ventilatory / oxygen therapies. | This data is now included in table 1 and figure 3 |
| 3.5 | Abstract: Timing of COVID waves differed in many countries. Summary timings for each should also be provided in abstract. | done |
| 3.6 | Introduction: Paragraph about report on hospital acquired COVID not relevant? Intro should describe the knowledge gap to be addressed by this paper. The aim of the paper should be more clearly spelt out. | Removed  Expanded aim at end of introduction on ward based care |
| 3.7 | Methods: Mortality – clarify ‘in-hospital mortality’, ‘x-day mortality’, ‘case fatality’ etc | Clarified in-hospital mortality (line 112) |
| 3.8 | Methods: Missing data – More information should be provided about how imputation was done. What was imputed and how? | See 2.13 |
| 3.9 | Methods: How many were missing mortality outcome data? These should just be excluded and reported as missing, not undergo imputation of outcomes. | No records were missing outcome at discharge. Mortality was a mandatory data field |
| 3.10 | Methods: The setting should be more clearly described. This is important for an international audience. Authors state data collection occurred at all 18 hospitals but report 6 health boards. For non-UK readers such as myself, it would help to explain what an HB is. I presume this is an organisational grouping of more than one hospital. If so, how many hospitals per HB? | Brief description of HB included (lines 117-8) |
| 3.11 | Methods: Description of the subgroups examined should be much clearer. It looks like they have examined all hospital admissions, then performed subgroup analyses of those who were admitted to the ward (as opposed to ICU) with further stratification by therapy provided (oxygen and CPAP/HFNO). Standardised analyses should be provided and replicated for each subgroup. | Methods (and results) completely rewritten with this in mind |
| 3.12 | Methods: Is the aim to compare outcomes by HB? If yes, include their outcomes in the paper along with the demographics of each group. If not (which I would recommend), I suggest removing this HB-specific information to an appendix and just reporting the overall numbers for all hospitals for each wave in the main manuscript. | Table changed to all Wales data, with HB data for appendix |
| 3.13 | Methods: HFNO and CPAP are not the same therapy, and require different degrees of intervention, monitoring, nursing attendance and technical maintenance. Is there data to differentiate the two? Was pressure support / bilevel non-invasive ventilation provided and recorded? Has this been aggregated with the CPAP group? | Those on HFNO only have been removed from this analysis and results are displayed for these on ward and IVU (figure 3). Bilevel ventilation was not used, unless patients had concurrent ventilatory failure. We did not specifically record data on bilevel ventilation |
| 3.14 | Methods: The authors also provide information for the outcomes for those admitted to ICU as a figure and report the mortality of those who receive CPAP/HFNO in ICU in the text. This is confusing to follow, particularly given they do not provide information about how many receive invasive ventilation, or how many went on from requiting CPAP/HFNO to needing ICU admission with or without CPAP/HFNO or invasive ventilation. How were patients classified if they required CPAP both in the ward and in ICU (latter presumably)? How many patients in each of these groups required invasive mechanical ventilation. | We have amended the analysis (table 1 and figure 2) to include this information. We recorded patients who required CPAP on wards then ICU as ICU as we utilised the highest level of care |
| 3.15 | Results: The presentation of results is disorganised. I would suggest presenting the result for the whole study cohort, then the comparison of the waves for the whole study cohort, then reporting the subgroups (which need to be more clearly delineated in the Methods section). | Rewritten completely as suggested |
| 3.16 | Results: More information should be provided about inclusions and exclusions. How were patients who were admitted to hospital more than once dealt with? Why were patients with LOS < 1 day excluded? Did this result in the exclusion of early deaths also? Where were the remaining 60% of the 14,614 patients? An inclusions/exclusions flow chart should be provided. | We only looked at index admission, and therefore would have missed readmission (added to limitations line 335).  We excluded those discharged with LOS <1 day, because these were a less severe group who were deemed not to require hospitalisation.  Our estimate of 40% of all cases is an underestimate. The denominator data we requested from Public Health Wales explicitly stated those aged >18, with LOS>1 day. We had to remove 8.6% of these datasets, so our data collection is closer to 45% of all cases. Data collection was a big resource utilisation involving junior doctors doing this and this largely limited data input possibilities |
| 3.17 | Results: Missingness is provided for some variables in the supplement but not for many of the other variables reported in the main manuscript. | Missingness in supplementary table |
| 3.18 | Results: Is there information about how long patients spent in the hospital? | In text and supplementary table |
| 3.19 | Results: Line 133 – ‘datasets’ do the authors mean observations in their dataset or that there were actually 6444 datasets? | We mean single patient datasets. |
| 3.20 | Results: Table 1 – There is non-standard reporting of proportions – better as n (%). There is no table of overall outcomes. This should be provided for all patients, those in each wave and for other relevant subgroups. | n for each wave is in table 1 and to put each n with each respective % would mean repeating each n 4 times. |
| 3.21 | Results: The numbers went up in some HBs and down in others over the 3 waves. Is this a consequence of the waves affecting hospitals/regions differently or were there differences in data collection or missingness which affected this? | It is both. The numbers were highest in Wave 2. Different hospitals had variable success in collecting data (which generally relied on junior doctors working out of hours for no extra pay to input data) |
| 3.22 | Results: Mortality and numbers for those admitted to ICU are shown in figure 1, but I can’t see where the actual numbers of patients admitted to ICU are described. Given mortality is the main outcome measure, the actual values should also be provided in addition to the figure. | Addressed as discussed with table 1 |
| 3.23 | Results: Fig 1 – ICU mortality - Clarify what this is: ‘in-ICU mortality’ or ‘in-hospital mortality of all patients admitted to ICU’ or ‘case fatality rate of any patient with COVID admitted to ICU’ etc | In hospital mortality of all patients who went to ICU |
| 3.24 | Results: Fig 2 and 3 look almost exactly the same. While I recognise the authors state that multivariable adjustment had little influence on the results of univariate logistic regression, I am surprised by how little change there is. I can’t see any change for most variables. Can this please be checked? Could high levels of imputation have affected this? | Univariate in supplementary. This has been checked and is true to the data |
| 3.25 | Results: Authors state frailty data was recorded but none is reported in the main paper. This would be an important risk adjustor to include in the main analysis if possible. Frailty completeness data is provided in the appendix but it is not clear if this is for all admissions, over 65s, nor what frailty score has been used and how this should be interpreted. | Frailty was recorded, but not well. We used a Rockwood frailty score which is only validated in over 65s. 38.8% of all patients over 65 had a frailty score recorded. Moreover, only a minority of patients in ICU were over 65 (only 28% of those on CPAP in ICU. Therefore, we did not use frailty data |
| 3.26 | Results: Table 1 is difficult to read and should be reformatted. See earlier comment about reporting by HB also, and consider removing this information from the table in the main manuscript. | Table 1 completely changed |
| 3.27 | Results: Does figure 5 refer to CPAP alone or CPAP and HFNO? If the latter, then differences in the proportion of patients who received CPAP or HFNO between ICU and the ward might explain their results. This is before even considering the competing events of invasive ventilation (or death). | HFNO only removed from this group on wards and ICU and considered separately |
| 3.28 | Results: Do the authors have information on time spent receiving each of therapies? | Added to results and supplementary table |
| 3.29 | Results: Adjusted results are provided for CPAP in ICU vs ward, but no raw mortality. The full adjusted analysis should be provided in table form for any primary outcome assessed in the whole group or in a subgroup. | now in table 1 |
| 3.30 | Results: Have the authors accounted for between site variation in outcomes affecting their overall results, and how completeness of information at each site might have affected their study? E.g mixed effects modelling, site/HB as RE? | This was considered and added to results section |
| 3.31 | Discussion: The discussion might be better presented in a more standard way, where all results are summarized briefly at the beginning, followed by comparison to literature and then putting this together with implications of the study. | Significant changes have been made to address this |
| 3.32 | Discussion: PCR testing – were pick-up diagnostic rates good during the first wave? Could cases have been missed and if so could these have affected your findings? | All in-patients were routinely tested throughout the time of the study, so we don’t believe that there were missed cases. |
| 3.33 | Discussion: The authors state that a strength is that this was “… a truly national…” dataset. However information on 60% of COVID admissions is missing. Can this be truly nationally representative? | We are not aware of many studies that have collected data over multiple waves and from all hospitals at a national level. We underestimate the proportion which is closer to 45% as the denominator data was inaccurate. The resources available limited the ability to access more notes and we feel that a dataset of 5887 patients is significant |
| 3.34 | Discussion: Having complete data for mandatory fields - is this a strength? This is exactly what I would expect for mandatory data fields. | Indeed, but the nature of the online data collection tool meant that mandatory fields had to be filled in or the entry could not be completed. Mandatory fields in paper collection tools are not always completed |
| 3.35 | Discussion: The inclusion of community acquired COVID is also not a specific strength of the study. This is just their study group. Their statement makes it sound like they are pleased they have made their mortality numbers look better than they really are by excluding hospital acquired COVID. If truly reporting the mortality associated with COVID-19, then all cases should be included. Thus I think this is a limitation of their study, not a strength. | Agree with this comment and this has been removed, and addressed as a limitation |
| 3.36 | Discussion: The authors mix comparisons with literature into their strengths and limitations sections. | Agree, this has been changed so strengths and limitations are separate short paragraphs |
| 3.37 | Minor points: Line 133 - ‘missing data imputation’ no capitalisation of M. | That’s because there should have been a “No” in front of it. |
| 3.38 | Minor points: Line 147 – ‘Mortality rates and admission numbers for all patients was…’ should be ‘…were…’ | changed |
| 3.39 | Minor points: Authors should avoid subjective language and use simpler statements which summarise data which can be read from the tables & figures. E.g. “There was a clear effect of age with significantly lower odds ratios in all ages before 70-79 and significantly higher odds ratios for ages from 80 onwards.” The finding would be the same irrespective of which reference group was used and could simply be stated with something like “Increasing age was associated with progressively higher mortality.” |  |
| 3.40 | Minor points: Capitalisation of waves should be checked through for consistency whether using a nominative ‘Wave n’ or just describing waves (which is sometimes capitalised and sometimes not). | Changed to Wave |
| 3.41 | Minor points: ‘…on ICU’ – very English English from the Welsh! ‘…in ICU’ is standard terminology for the rest of the world. Please change. | OK, fair enough, changed |
| 3.42 | Minor points: COVID should be COVID-19 throughout the manuscript. | changed |

**Original comments**

Journal Requirements:

When submitting your revision, we need you to address these additional requirements.

1. Please ensure that your manuscript meets PLOS ONE's style requirements, including those for file naming. The PLOS ONE style templates can be found at

[https://journals.plos.org/plosone/s/file?id=wjVg/PLOSOne_formatting_sample_main_body.pdf](https://scanmail.trustwave.com/?c=261&d=wNTb5Boroe-Et8blfvKtoZRAFhBLa_LNvlqFO1WKjg&u=https%3a%2f%2fjournals%2eplos%2eorg%2fplosone%2fs%2ffile%3fid%3dwjVg%2fPLOSOne%5fformatting%5fsample%5fmain%5fbody%2epdf) and

[https://journals.plos.org/plosone/s/file?id=ba62/PLOSOne_formatting_sample_title_authors_affiliations.pdf](https://scanmail.trustwave.com/?c=261&d=wNTb5Boroe-Et8blfvKtoZRAFhBLa_LNvluHawOJhg&u=https%3a%2f%2fjournals%2eplos%2eorg%2fplosone%2fs%2ffile%3fid%3dba62%2fPLOSOne%5fformatting%5fsample%5ftitle%5fauthors%5faffiliations%2epdf)

2. We note that the grant information you provided in the ‘Funding Information’ and ‘Financial Disclosure’ sections do not match.

When you resubmit, please ensure that you provide the correct grant numbers for the awards you received for your study in the ‘Funding Information’ section.

3. Thank you for stating the following financial disclosure:

"Welsh Government Funds the Respiratory Health Implementation Group (RHIG) and Respiratory Innovation Wales. RHIG fund the Institute for Clinical Science and Technology (ICST), who create and implement the digital innovations."

Please state what role the funders took in the study.  If the funders had no role, please state: "The funders had no role in study design, data collection and analysis, decision to publish, or preparation of the manuscript."

If this statement is not correct you must amend it as needed.

Please include this amended Role of Funder statement in your cover letter; we will change the online submission form on your behalf.

3. Thank you for stating the following in your Competing Interests section:

“no authors have competing interests”

Please complete your Competing Interests on the online submission form to state any Competing Interests. If you have no competing interests, please state "The authors have declared that no competing interests exist.", as detailed online in our guide for authors at [http://journals.plos.org/plosone/s/submit-now](http://scanmail.trustwave.com/?c=261&d=wNTb5Boroe-Et8blfvKtoZRAFhBLa_LNvg2IbADcjg&u=http%3a%2f%2fjournals%2eplos%2eorg%2fplosone%2fs%2fsubmit-now).

 This information should be included in your cover letter; we will change the online submission form on your behalf.

4. Please include captions for your Supporting Information files at the end of your manuscript, and update any in-text citations to match accordingly. Please see our Supporting Information guidelines for more information: [http://journals.plos.org/plosone/s/supporting-information](http://scanmail.trustwave.com/?c=261&d=wNTb5Boroe-Et8blfvKtoZRAFhBLa_LNvguEbwyO1w&u=http%3a%2f%2fjournals%2eplos%2eorg%2fplosone%2fs%2fsupporting-information).

[Note: HTML markup is below. Please do not edit.]

Reviewers' comments:

Reviewer's Responses to Questions

**Comments to the Author**

1. Is the manuscript technically sound, and do the data support the conclusions?

The manuscript must describe a technically sound piece of scientific research with data that supports the conclusions. Experiments must have been conducted rigorously, with appropriate controls, replication, and sample sizes. The conclusions must be drawn appropriately based on the data presented.

Reviewer #1: Yes

Reviewer #2: Yes

Reviewer #3: Partly

2. Has the statistical analysis been performed appropriately and rigorously?

Reviewer #1: Yes

Reviewer #2: Yes

Reviewer #3: No

3. Have the authors made all data underlying the findings in their manuscript fully available?

The [PLOS Data policy](http://scanmail.trustwave.com/?c=261&d=wNTb5Boroe-Et8blfvKtoZRAFhBLa_LNvgGBPgbc0w&u=http%3a%2f%2fwww%2eplosone%2eorg%2fstatic%2fpolicies%2eaction%23sharing) requires authors to make all data underlying the findings described in their manuscript fully available without restriction, with rare exception (please refer to the Data Availability Statement in the manuscript PDF file). The data should be provided as part of the manuscript or its supporting information, or deposited to a public repository. For example, in addition to summary statistics, the data points behind means, medians and variance measures should be available. If there are restrictions on publicly sharing data—e.g. participant privacy or use of data from a third party—those must be specified.

Reviewer #1: Yes

Reviewer #2: Yes

Reviewer #3: Yes

4. Is the manuscript presented in an intelligible fashion and written in standard English?

PLOS ONE does not copyedit accepted manuscripts, so the language in submitted articles must be clear, correct, and unambiguous. Any typographical or grammatical errors should be corrected at revision, so please note any specific errors here.

Reviewer #1: Yes

Reviewer #2: Yes

Reviewer #3: Yes

5. Review Comments to the Author

Please use the space provided to explain your answers to the questions above. You may also include additional comments for the author, including concerns about dual publication, research ethics, or publication ethics. (Please upload your review as an attachment if it exceeds 20,000 characters)

Reviewer #1: Dear authors,

Thank you for the opportunity to review this nationwide, retrospective analysis of hospitalized patients with Covid-19.

Data was collected though an online, anonymised database and the project was assessed by an ethical review board but no opinion was required due to the nature of the study.

Title: Reflects to contents of the study

Abstract: Straigth to the point. Reads well. Aim, findings and conclusion aligned.

Introduction:

LL43-50: The authors make a huge point of the national guideline. This part is quite unexpected and please justify why this part is required, as it does not seem to be relevant to the data presented. Or was the guideline and database tighly connected? Please clarify.
L50: The last meaning on avoiding invasive mechanical ventilation whenever possible spikes an interest. This strategy is similar to what was experienced from northern Italy. In light of your results, I think your viewpoint regarding this practice would be quite interesting. Did the strategy change over time when pressure on ICU/ventilator beds decreased?

Method:
L967: Please specify when mortiality was assessed. During index admission? Hospital mortality? 30d? If patients were discharged to for example a nursing facility and succumbed shortly thereafter, was this registered as an primary event outcome? Please specify.

Statistical analysis: Appears to be sound.

Results:
Well presented, Fig 1 and Fig 2 are informative and yields relevant information.

Just out of curiosity, why did you chose the specific baseline/reference categories? Eg Wave 2 as reference instead of 1 or 3? (No need to revise!)

Discussion:

LL216-218: Consider omitting this, the aim was already stated. I would rather see your major findings first in the discussion.
LL227-228: I would add that the reduction in mortality over was was likely due to.... There is no data to support increasing experience - for pharmaceuticals, pls refer to the supplement table.
LL230-233: Does your data support this?
LL236-240: I think this is a v imp finding - consider adding that this could be attributed to the publicly funded healthcare system (if you agree of course!)
LL255-265: Here you pick up on the topic I commented on earlier - I would like to see a deeper analysis. In hindsight, would you do the same choice again? This is important for future desicion-making! Did the propotion of patients on invasive ventilation change over time?
LL282: Consider adding that less than half the hospitalised patients were registered to the database.


I enjoyed reading this report and I think it is valuable for future decision-making and hoping that my feedback could help improving the report further.

Reviewer #2: Thank you for the opportunity to review this interesting work. Data are limited on the association between sociodemographic status and outcomes as well as the comparison of outcomes by wards and receipt of respiratory care.

Please find below suggested comments for consideration:

1. Data are plural and the text should be updated accordingly (e.g. line 20 “data were collected” rather than “data was collected”)
2. Abstract, methods – line 23, please define adults (> 18 years).
3. Abstract, methods – suggest including a sentence regarding the statistical analyses performed.
4. Abstract, results – suggest including the number of hospitals
5. Ensure that “COVID-19” is used throughout the text rather than “COVID” (e.g. lines 50, 58 etc.)
6. Methods, line 65 – suggest including additional information to describe the acute care hospitals if available (e.g. average bed size, adult vs. pediatric sites, teaching vs. non-teaching hospitals)
7. Methods, line 69 – please spell out HB the first time it is referenced
8. Methods, line 76 “continuous notes review by local clinical teams” – suggest specifying or describing what this means. Does it mean that admissions records were checked to see which ones had clinician notes available? Please clarify.
9. Methods – suggest spelling out CPAP and defining HFNO which is referenced in the results but not in the methods
10. Methods, lines 85-87 – suggest providing additional details regarding WIMD, specifically defining the levels or categories of deprivation status (e.g. 50% most deprived).
11. Methods, line 96 – please define mortality. It is all-cause in-hospital mortality? 30-day mortality?
12. Methods, lines 102-103 – if possible, suggest indicating why some post codes did not link to a deprivation status (e.g. outside of geographic area of study? Or not mapped to status?)
13. Methods, line 105 – if possible, please provide further information regarding how missing data imputation was used.
14. Methods, line 105-106 – to confirm was BMI excluded from the analysis due to incomplete records? Please clarify
15. Methods, line 109 – Health Boards is spelled out here. For consistency, suggest spelling out the first time it is used than consistently using HB.
16. Results, line 133 – lower case “wave”, for consistency suggest using capital “Wave”.
17. Results, lines 133-135 – the term patients, datasets and patient dataset are used interchangeably here. Suggest selecting one term to use consistently.
18. Results, lines 138-139 – is this referencing the median or average age? Please clarify. And “those admitted in Wave 3 were 4 years younger” – the median or average age was 4 years younger?
19. Results, lines 139-140 “likely reflecting increased vaccination rates in the older age groups protecting against admission” – this is not a result but better suited in the discussion. Also, are there data to support this statement? Are population level age-stratified vaccination rates available?
20. Table 1 – consider adding p value results.
21. Results, lines 152-159 – suggest to include key relevant data (e.g. odds ratios and 95% confidence intervals) either in the text or figures.
22. Results, lines 167-175 – as above, helpful to have odds ratios and 95% CI reported here for key results.
23. Results, lines 167-175 – suggest reporting as “odds of death” (e.g. the effect of age persisted with a lower odds of death at lower age…”)
24. Results, lines 193-195 – suggest confirming that this difference is statistically significant? Regardless, is it clinically significant? A difference of 1 comorbidity?
25. Results, lines 208-209 – suggest adding definition of fully vaccinated in methods. Also, suggest clarifying sentence to “The overall mortality among unvaccinated patients was 20.5%, compared to 4.8% among patients who were fully vaccinated.”
26. Results, lines 211-212 – suggest comparing to the same group (e.g. 81% were unvaccinated compared to 26.6% unvaccinated). The comparison of unvaccinated to vaccinated is not useful.
27. Discussion, line 219 – the data represents approximated 40% of total CA COVID admissions in acute hospitals in Wales? Of do the data represent 40% of all admissions (COVID and non-COVID)? Perhaps clarify because above it is stated that every acute hospital in Wales is captured in these data.
28. Discussion, line 223 – suggested updating to “our patients had higher mortality associated with increasing…”
29. Discussion, line 247 – suggest using consistent terminology “fully vaccinated” vs. “double vaccinated with a booster”
30. Discussion, line 247 – hesitant to use the word “confirm” here as this study did not control for other factors which may have either contributed to death (e.g. underlying comorbidities, age etc.) or improved chance of survival (such as age or prior/natural immunity)
31. Discussion, lines 247-248 – cannot conclude that vaccines protect against hospitalization as this was not measured in this study (you would need community level data to compare to those CA COVID-19 positive not hospitalized by vaccination status).
32. Discussion, lines 266 – consider conducting a multivariable logistic regression to compare those who received CPAP and those who did not with the outcome of mortality to provide further support/strength this conclusion.
33. Figure 1 – should the grey bar be “non ICU admissions”?

Reviewer #3: Re: PONE-D-23-05105
COVID-19 Managed on Respiratory Wards and Intensive Care Units: Results from the National COVID-19 Outcome Report in Wales From March 2020 to December 2021

Thank you for asking me to review this retrospective study of 5887 patients with COVID-19 admitted to all 18 acute hospitals in Wales between March 2020 and December 2021. The authors report declining mortality for those requiring oxygen or CPAP over the pandemic period with stepwise reduction over successive waves from 1 to 3. They also report no difference in mortality outcomes between those who received CPAP in wards as opposed to ICU.

The attempt to standardise management across Welsh hospitals and collect data about this was laudable. There is a lot of potentially interesting data here but the presentation of the results is disorganised. The paper lacks a clear aim. The paper falls between a scientific paper aiming to address a specific research question and a generic health department style report covering multiple aspects of care provided to COVID patients. If the aim is the former, it should be structured to address a specific question (e.g. change in mortality over the 3 waves). If the aim is the latter, it should be structured as a report with more complete ordered information with less commentary, although this then might be difficult to meet the requirements for publication as a scientific paper, and would be better suited to an on-line health department report.

The authors have also focused in on a result from a subgroup (CPAP in ICU) which does not appear to be a primary aim of the paper. If the main aim is to determine the how outcomes differ between patients who are treated with CPAP in the ward as opposed to ICU, then there should be a much more robust analysis to back up their findings, and this should become the focus of the paper. (See later comment also).

Outcomes from respiratory wards and ICUs are reported. Does this mean that patients admitted to other wards are not included? What happened to COVID patients put on other wards or have all patients admitted with COVID been included in the study?

Do the authors have information on how many patients received invasive ventilation? This is vital to interpret the prevalence and outcomes associated with provision of other respiratory / ventilatory / oxygen therapies.

Specific comments

Abstract

Timing of COVID waves differed in many countries. Summary timings for each should also be provided in abstract.

Introduction

Paragraph about report on hospital acquired COVID not relevant? Intro should describe the knowledge gap to be addressed by this paper.
The aim of the paper should be more clearly spelt out.

Methods

Mortality – clarify ‘in-hospital mortality’, ‘x-day mortality’, ‘case fatality’ etc

Missing data – More information should be provided about how imputation was done. What was imputed and how?

How many were missing mortality outcome data? These should just be excluded and reported as missing, not undergo imputation of outcomes.

The setting should be more clearly described. This is important for an international audience. Authors state data collection occurred at all 18 hospitals but report 6 health boards. For non-UK readers such as myself, it would help to explain what an HB is. I presume this is an organisational grouping of more than one hospital. If so, how many hospitals per HB?

Description of the subgroups examined should be much clearer. It looks like they have examined all hospital admissions, then performed subgroup analyses of those who were admitted to the ward (as opposed to ICU) with further stratification by therapy provided (oxygen and CPAP/HFNO). Standardised analyses should be provided and replicated for each subgroup.

Is the aim to compare outcomes by HB? If yes, include their outcomes in the paper along with the demographics of each group. If not (which I would recommend), I suggest removing this HB-specific information to an appendix and just reporting the overall numbers for all hospitals for each wave in the main manuscript.

HFNO and CPAP are not the same therapy, and require different degrees of intervention, monitoring, nursing attendance and technical maintenance. Is there data to differentiate the two? Was pressure support / bilevel non-invasive ventilation provided and recorded? Has this been aggregated with the CPAP group?

The authors also provide information for the outcomes for those admitted to ICU as a figure and report the mortality of those who receive CPAP/HFNO in ICU in the text. This is confusing to follow, particularly given they do not provide information about how many receive invasive ventilation, or how many went on from requiting CPAP/HFNO to needing ICU admission with or without CPAP/HFNO or invasive ventilation. How were patients classified if they required CPAP both in the ward and in ICU (latter presumably)? How many patients in each of these groups required invasive mechanical ventilation.

Results

The presentation of results is disorganised. I would suggest presenting the result for the whole study cohort, then the comparison of the waves for the whole study cohort, then reporting the subgroups (which need to be more clearly delineated in the Methods section).

More information should be provided about inclusions and exclusions. How were patients who were admitted to hospital more than once dealt with? Why were patients with LOS < 1 day excluded? Did this result in the exclusion of early deaths also? Where were the remaining 60% of the 14,614 patients? An inclusions/exclusions flow chart should be provided.

Missingness is provided for some variables in the supplement but not for many of the other variables reported in the main manuscript.

Is there information about how long patients spent in the hospital?

Line 133 – ‘datasets’ do the authors mean observations in their dataset or that there were actually 6444 datasets?

Table 1 – There is non-standard reporting of proportions – better as n (%). There is no table of overall outcomes. This should be provided for all patients, those in each wave and for other relevant subgroups.

The numbers went up in some HBs and down in others over the 3 waves. Is this a consequence of the waves affecting hospitals/regions differently or were there differences in data collection or missingness which affected this?

Mortality and numbers for those admitted to ICU are shown in figure 1, but I can’t see where the actual numbers of patients admitted to ICU are described. Given mortality is the main outcome measure, the actual values should also be provided in addition to the figure.

Fig 1 – ICU mortality - Clarify what this is: ‘in-ICU mortality’ or ‘in-hospital mortality of all patients admitted to ICU’ or ‘case fatality rate of any patient with COVID admitted to ICU’ etc

Fig 2 and 3 look almost exactly the same. While I recognise the authors state that multivariable adjustment had little influence on the results of univariate logistic regression, I am surprised by how little change there is. I can’t see any change for most variables. Can this please be checked? Could high levels of imputation have affected this?

Authors state frailty data was recorded but none is reported in the main paper. This would be an important risk adjustor to include in the main analysis if possible. Frailty completeness data is provided in the appendix but it is not clear if this is for all admissions, over 65s, nor what frailty score has been used and how this should be interpreted.

Table 1 is difficult to read and should be reformatted. See earlier comment about reporting by HB also, and consider removing this information from the table in the main manuscript.

Does figure 5 refer to CPAP alone or CPAP and HFNO? If the latter, then differences in the proportion of patients who received CPAP or HFNO between ICU and the ward might explain their results. This is before even considering the competing events of invasive ventilation (or death).

Do the authors have information on time spent receiving each of therapies?

Adjusted results are provided for CPAP in ICU vs ward, but no raw mortality. The full adjusted analysis should be provided in table form for any primary outcome assessed in the whole group or in a subgroup.

Have the authors accounted for between site variation in outcomes affecting their overall results, and how completeness of information at each site might have affected their study? E.g mixed effects modelling, site/HB as RE?

Discussion

The discussion might be better presented in a more standard way, where all results are summarized briefly at the beginning, followed by comparison to literature and then putting this together with implications of the study.

PCR testing – were pick-up diagnostic rates good during the first wave? Could cases have been missed and if so could these have affected your findings?

The authors state that a strength is that this was “… a truly national…” dataset. However information on 60% of COVID admissions is missing. Can this be truly nationally representative?

Having complete data for mandatory fields - is this a strength? This is exactly what I would expect for mandatory data fields.

The inclusion of community acquired COVID is also not a specific strength of the study. This is just their study group. Their statement makes it sound like they are pleased they have made their mortality numbers look better than they really are by excluding hospital acquired COVID. If truly reporting the mortality associated with COVID-19, then all cases should be included. Thus I think this is a limitation of their study, not a strength.

The authors mix comparisons with literature into their strengths and limitations sections.

Minor points

There are grammatical mistakes which need a thorough read through and check

Line 133 - ‘missing data imputation’ no capitalisation of M.

Line 147 – ‘Mortality rates and admission numbers for all patients was…’ should be ‘…were…’

Authors should avoid subjective language and use simpler statements which summarise data which can be read from the tables & figures. E.g. “There was a clear effect of age with significantly lower odds ratios in all ages before 70-79 and significantly higher odds ratios for ages from 80 onwards.” The finding would be the same irrespective of which reference group was used and could simply be stated with something like “Increasing age was associated with progressively higher mortality.”

Capitalisation of waves should be checked through for consistency whether using a nominative ‘Wave n’ or just describing waves (which is sometimes capitalised and sometimes not).

‘…on ICU’ – very English English from the Welsh! ‘…in ICU’ is standard terminology for the rest of the world. Please change.

COVID should be COVID-19 throughout the manuscript.
